# Subject–Motion Correction in HARDI Acquisitions: Choices and Consequences

**DOI:** 10.3389/fneur.2014.00240

**Published:** 2014-12-09

**Authors:** Shireen Elhabian, Yaniv Gur, Clement Vachet, Joseph Piven, Martin Styner, Ilana R. Leppert, G. Bruce Pike, Guido Gerig

**Affiliations:** ^1^Scientific Computing and Imaging Institute, Salt Lake City, UT, USA; ^2^Faculty of Computers and Information, Cairo University, Cairo, Egypt; ^3^IBM Almaden Research Center, San Jose, CA, USA; ^4^Department of Psychiatry, University of North Carolina, Chapel Hill, NC, USA; ^5^Department of Computer Science, University of North Carolina, Chapel Hill, NC, USA; ^6^Department of Neurology and Neurosurgery, Montreal Neurological Institute, Montreal, QC, Canada; ^7^Department of Radiology, University of Calgary, Calgary, AB, Canada

**Keywords:** HARDI, subject motion, motion correction, fiber orientations, orientation distribution functions, tractography comparison, impact quantification

## Abstract

Diffusion-weighted imaging (DWI) is known to be prone to artifacts related to motion originating from subject movement, cardiac pulsation, and breathing, but also to mechanical issues such as table vibrations. Given the necessity for rigorous quality control and motion correction, users are often left to use simple heuristics to select correction schemes, which involves simple qualitative viewing of the set of DWI data, or the selection of transformation parameter thresholds for detection of motion outliers. The scientific community offers strong theoretical and experimental work on noise reduction and orientation distribution function (ODF) reconstruction techniques for HARDI data, where post-acquisition motion correction is widely performed, e.g., using the open-source DTIprep software (1), FSL (the FMRIB Software Library) (2), or TORTOISE (3). Nonetheless, effects and consequences of the selection of motion correction schemes on the final analysis, and the eventual risk of introducing confounding factors when comparing populations, are much less known and far beyond simple intuitive guessing. Hence, standard users lack clear guidelines and recommendations in practical settings. This paper reports a comprehensive evaluation framework to systematically assess the outcome of different motion correction choices commonly used by the scientific community on different DWI-derived measures. We make use of human brain HARDI data from a well-controlled motion experiment to simulate various degrees of motion corruption and noise contamination. Choices for correction include exclusion/scrubbing or registration of motion corrupted directions with different choices of interpolation, as well as the option of interpolation of all directions. The comparative evaluation is based on a study of the impact of motion correction using four metrics that quantify (1) similarity of fiber orientation distribution functions (fODFs), (2) deviation of local fiber orientations, (3) global brain connectivity via graph diffusion distance (GDD), and (4) the reproducibility of prominent and anatomically defined fiber tracts. Effects of various motion correction choices are systematically explored and illustrated, leading to a general conclusion of discouraging users from setting *ad hoc* thresholds on the estimated motion parameters beyond which volumes are claimed to be corrupted.

## 1 Introduction

Diffusion-weighted (DW)-MRI enables probing the fiber architecture of biological tissues – *in vivo* – by encoding the microscopic direction and speed of the diffusion of water molecules (4), while reflecting the amount of hindrance experienced by such molecules along the axis of the applied diffusion gradient due to barriers and obstacles imposed by micro-structures (5). Today, diffusion tensor imaging (DTI) is the method of choice for most neuroimaging studies, e.g., autism (6), schizophrenia (7), and Huntington’s disease (8). Nonetheless, DTI assumes a homogeneous axon population inside a single voxel (9) and fails at modeling more realistic heterogeneous populations. High angular resolution diffusion imaging (HARDI) (10), on the other hand, allows the diffusion acquisition to focus on the angular component of the DW signal using strong gradients and long diffusion times (5), while revealing the intra-voxel orientational heterogeneity, such as crossing and merging fiber bundles. The promising potential of HARDI-based DW-MRI in describing fiber tracts within the human brain comes with a price tag of a wide variety of artifacts related to the gradient system hardware, pulse sequence, acquisition strategy, and subject motion (11). Such artifacts render the quality of diffusion imaging questionable and reduce the accuracy of findings when left uncorrected (1).

### 1.1 Motion artifacts

In today’s clinical DW-MRI acquisitions, the presence of the long and strong gradient pulses have made diffusion MRI more sensitive to the detrimental effects of subject motion than other MRI techniques (9, 12, 13). During a scanning session, the degree of a patient’s cooperation may vary: elderly people who may become uncomfortable during large scanning sessions, patients in pain who become restless and agitated during a scan, and unsedated pediatric subjects who will not cooperate long enough to be imaged without motion artifacts. Hence, it is safe to assume that there are always motion artifacts in any given DW-MRI acquisition due to the increased likelihood of involuntary subject motion; especially with HARDI acquisitions, which use a large number of gradient directions resulting in longer scan times. A proof-of-concept of this hypothesis is presented in section 1.

Motion artifacts range from physiological motion (e.g., cardiac pulsation and respiration) to physical (voluntary or involuntary) *bulk movement* by the patient (14). Physiological motion can be controlled by gating or in the sequence design (15), but the patient bulk movement during the diffusion-encoding gradient pulses leads to severe signal perturbation (16–18), which results in a significant signal phase shift or signal loss (19). The effects of bulk motion are twofold: *slow bulk motion* can cause misalignment of diffusion data between subsequent gradient applications (i.e., DWI-volumes), resulting in an underestimation of diffusion anisotropy (4), whereas *fast bulk motion* during the application of a single diffusion gradient causes inhomogeneous signal dropout/attenuation artifacts in the diffusion-weighted images. This dropout effect arises due to signal dephasing within the voxels (13, 14), which is the very phenomenon that gives rise to the DW-MRI contrast, leading to an overestimation of diffusion anisotropy (4). Although misalignment can be tackled by registration-based correction methods (20), the signal dropout due to intragradient motion will persist (4), where such images are identified and excluded from further processing and/or scheduled for reacquisition during the same scan (13, 14, 21–23). Left uncorrected, motion-corrupted datasets introduce bias in the subsequent findings due to the induced variability of diffusion MRI measurements, while affecting the statistical properties of diffusion derived measures in heterogeneous brain regions.

### 1.2 Motion correction choices

The identification and elimination of slow bulk motion artifacts in HARDI data, which are characterized by a high b-value and low signal-to-noise (SNR) ratio, still remains a challenge. In order to allow correction approaches to proceed with reasonable accuracy, motion occurring between diffusion gradients can be treated as if it occurred all at once (24).

Motion effects can be reduced by real-time motion control during the acquisition (a.k.a. *prospective* motion correction) (25–27), where the acquisition and the source of motion are synchronized, so that the data are never corrupted. In addition, the development of accelerated acquisition methods [e.g., Ref. (28)] can reduce the duration of a scan to minimize the susceptibility of subject motion. A comfortable padding can also be used to minimize head motion while urging the participant to remain without movement (11). Nonetheless padding is not always effective in studies involving infants [e.g., autism diagnosis (29)], where remaining still in the scanner may be more challenging. Nevertheless, prospective methods for motion correction might affect the acquisition time due to the reacquisition of motion-corrupted gradients (14). Such methods might also require external optical tracking systems (23), free-induction decay navigators (26), or volumetric navigators (30), which are not always available on current scanners (27), coupled with the need of time-consuming calibration steps prior to their use (14). Furthermore, rapid modification of diffusion gradients may induce eddy current artifacts (13), and there is no guarantee that the head will move back to the original position.

Motion compensation can also be performed as a post-processing step after acquisition, i.e., *retrospective*, to guarantee voxel-wise correspondence between different DWIs referring to the same anatomical structure. A common practice is to heuristically select transformation parameter thresholds for detection of motion outliers, where registration and interpolation are applied to gradient directions that are claimed to be corrupted. Software packages for image-based registration of DWIs are becoming readily available, e.g., FSL-MCFLIRT (2, 31), the Advanced Normalization Tools (ANTS) (32), TORTOISE (3), and BRAINSFit (33) employed in DTIPrep (1).

A typical retrospective motion correction algorithm involves two stages (20): first, finding the global transformation parameters that would transform all DWIs to the same coordinate frame, and then, applying the estimated transformations to the diffusion data. Solving for the transformation parameters usually involves rigidly registering the DWIs to a reference volume representing the same anatomical structure, but without being contaminated by motion artifacts. Examples of such a reference include a T2-weighted image (16), or a non-diffusion-weighted image (a.k.a baseline with b-value = 0) due to its high SNR and lesser vulnerability to eddy current distortion (34), where the difference in intensity profiles is compensated for using normalized mutual information similarity measure. Another alternative is a model-based reference volume computed for each diffusion-weighted image based on tensor fitting (35, 36). Model-based motion correction implicitly assumes that the original position defined by the baseline volume is the reference position to be aligned to Sakaie and Lowe (20). Recently, it has been shown that model-based motion correction becomes a more powerful choice for correcting higher b-value diffusion imaging, which does not contain enough anatomical features to be registered accurately (36).

Applying the estimated transformation parameters is performed using *interpolation*, which computes intensities at transformed voxel coordinates as a weighted sum of the scaled intensities at surrounding voxels. The diffusion gradient vectors are also reoriented to incorporate the rotational component of subject motion (37). Interpolation is usually carried out by an exact fit of a continuously defined model to discrete data samples. Nonetheless, this exact fit is less appropriate when data are noise-corrupted, since the model is forced to fit the noise too. Although using regularized interpolation can tackle noisy data, it is only preferable to applying denoising followed by standard interpolation under the assumption that the signal is a *stationary Gaussian process* (38); a situation that is not applicable for diffusion-weighted images, which are contaminated by Rician noise. Based on the central limit theorem, the (weighted) average of a large set of i.i.d. samples tends to follow a normal distribution. Thus, interpolation between Rician distributed samples might change the distribution toward a Gaussian PDF (39). We can, therefore, argue that the denoising process decreases the effect of standard interpolation on altering the underlying data distribution.

Another retrospective approach is to cast motion correction as an outlier rejection process, ranging from simply excluding one or more gradients bearing strong motion artifacts beyond acceptable levels of motion (11, 14, 40), to statistical methods for detecting and discarding voxel-wise diffusion measurements as outliers (17, 41, 42). Usually discarding entire scans (a.k.a *motion scrubbing* in functional MRI) either can be performed by visual inspection or based on predefined thresholds on estimated motion parameters (4). Nevertheless, removing gradients limits the ability to reconstruct crossing fibers, especially at small separation angles, due to the decreased number of distinct gradient directions needed for diffusion reconstruction. Moreover, scrubbing would introduce intersubject SNR and bias differences that would in turn affect subsequent statistical analysis (1). On the other hand, local exclusion of corrupted voxels for robust diffusion reconstruction in the presence of outliers is based on the deviation of the observed measurements (usually after motion correction) from the assumed diffusion model. Using these approaches for motion correction itself would mingle the effect of being an outlier to an assumed model with that of being corrupted due to motion. Further, local exclusion would lead to a different number of DWIs locally available for each voxel, complicating subsequent analysis to avoid bias due to different SNR values for different brain regions (1).

A common concern with retrospective methods in clinical studies, whether registration-based and/or outlier-based, is that data with different levels of motion will be subject to different schemes of motion correction. For instance, patients may show more motion than controls, or sedated subjects may be different from non-sedated. Applying different motion correction schemes could introduce a confounding factor for statistical analysis of populations that show different motion patterns. Nonetheless, eyeballing the acquired/preprocessed DWIs prior to proceeding to further analysis is highly recommended.

### 1.3 Objective and contributions

The lack of a comprehensive/rigorous quality control (QC) for HARDI datasets can result in considerable error and bias in subsequent analyses, which may affect research studies using these datasets. Most current software packages such as DTIPrep (1), TORTOISE (3), and FSL (2), which offer various tools for processing and analysis of diffusion-weighted images, are mostly limited to DTI datasets, which are characterized by low b-values (i.e., higher SNR) and fewer gradients (i.e., shorter acquisition times). Nonetheless, special care is needed for HARDI datasets due to their low SNR and longer acquisition times, which increase the likelihood of subject motion. As a part of a thorough pipeline for HARDI-QC, this paper addresses the motion correction aspect for *slow bulk motion* where users often do not fully understand the consequences of different types of correction schemes on the final analysis, and whether those choices may introduce confounding factors when comparing populations. Therefore, the presented work is directed toward clear guidelines and recommendations to the standard users in practical settings.

**Figure 1:**
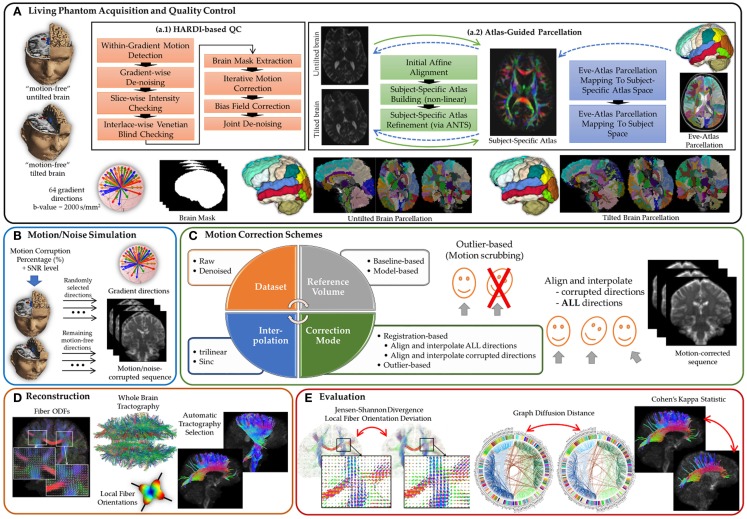
**A comprehensive experimental framework for subject motion simulation to systematically evaluate the outcome of different motion correction choices commonly used by the scientific community on HARDI-based reconstructions and tractography**. **(A)** A human brain HARDI data were acquired from a well-controlled motion experiment. (a.1) Acquired DWIs were preprocessed to obtain nearly noise-free and motion-free datasets. (a.2) For automated tractography selection and the quantification of whole brain connectivity, a subject-specific unbiased atlas was constructed via DTI-derived data from HARDI sequences resulting in a tensor atlas, where we can define a detailed parcelation of neuroanatomical structures, and map it back to each raw scan. **(B)** Noticeable motion was then simulated by randomly mixing gradients from the acquired datasets. **(C)** Motion correction involves four main decision variables where each distinct combination of choices defines a correction scheme. **(D)** Reconstruction of a corrected or motion-free dataset entails reconstructing the voxel-wise fiber orientation distribution functions, detecting local (voxel-wise) fiber orientation, preforming whole brain tractography, and automatically selecting anatomical pathways. **(E)** The evaluation of the effect of a motion correct scheme has been investigated based on voxel-wise metrics, global brain connectivity metric, and tract-based metric.

The optimal preprocessing pipeline for HARDI sequences remains an open question and a challenge for real data. Questions that might arise include: is there a threshold that would identify a motion-corrupted volume? How sensitive are HARDI reconstructions to such a predefined threshold? What is the impact of various motion correction schemes on subsequent HARDI-based reconstructions and tractography? So far, these questions have received, surprisingly, little attention in various DW-MRI studies of clinical populations. This study, then, focuses on the effect of preprocessing schemes, in particular motion correction, commonly deployed as a post-acquisition step, on succeeding steps. We propose a *comprehensive* experimental framework (see Figure 1) that enables making use of human brain HARDI data from a well-controlled motion experiment to simulate various degrees of motion/noise corruption. The comprehensiveness is related to the systematic evaluation of the outcome of different motion correction choices commonly used by the scientific community on different DWI-derived measures. To our knowledge, this evaluation does not exist in the literature and has not been discussed in detail.

Choices for correction include exclusion or registration of motion corrupted directions, with different choices of interpolation, as well as the option of registration/interpolation of all directions versus corrupted directions only. The effect of denoising as a preprocessing step applied prior to motion correction is also investigated. Further, the choice of the reference volume used in the registration framework is also discussed. The comparative evaluation covers four metrics: (1) the similarity of fiber orientation distribution functions (fODFs) via Jensen–Shannon divergence (JSD), (2) the deviation of multiple fiber orientations at each voxel, (3) the global brain connectivity via graph diffusion distance (GDD), and (4) the reproducibility of seven anatomically defined fiber pathways via Cohen’s Kappa statistics. On the basis of our findings, we recommend assuming that motion is inevitable, even subtle, in the acquired scans. Motion correction, therefore, needs to be applied to all gradient directions without relying heuristically on a threshold that determines a gradient direction to be claimed as motion corrupted.

## 2 Materials and Methods

### 2.1 Motion is inevitable: Proof-of-concept

To back up our assumption that motion is omnipresent, we analyzed data from three healthy human phantoms (males 30–40 years old) visiting each of the four clinical sites (Chapel Hill, Philadelphia, St. Louis, and Seattle) as a part of the ACE-IBIS study [Autism Centers for Excellence, Infant Brain Imaging study (6)], using a total of six MRI systems (two sites using both research and hospital scanners). All study procedures were approved by the institutional review board at each clinical site, and informed, written consent was obtained for all participants. In addition, the traveling phantoms sign consent forms at each of the sites, as per their own institutional IRBs. The sites include the University of Washington, Seattle, the Washington University in St. Louis, the Childrens hospital of Philadelphia, and the University of North Carolina at Chapel Hill. Each subject was scanned twice on a 3 T Siemens Tim Trio scanner^1^ with a strict calibration of image acquisition parameters. Test–retest reliability at each site was established with two scans within 24 h. The scans were acquired within 1 week to guarantee that there were no major brain changes over time. The scanning environment was well controlled. Comfortable padding was used to minimize head motion, and patients were urged to remain without movement. Eddy current was compensated for using a Twice Refocused Spin Echo (TRSE) protocol^2^, with FoV = 209 mm, 76 transversal slices, thickness = 2 mm (2 mm)^3^ voxel resolution, matrix size = 106 × 106, TR = 11100 ms, TE = 103 ms, one baseline image with zero b-value and 64 DWI with b-value at 2000 s/mm^2^, with a total scan time of 12.5 min.

Initially, we ran automated Quality Control on the DWIs via DTIPrep (1), which includes among other steps interlaced correlation analysis for detection and removal of fast bulk motion within a single DWI volume, where no quantitative within-gradient motion was detected. Inspired by Sakaie and Lowe (20), FSL-MCFLIRT (31) was then used to provide the rigid transformation matrix (i.e., six degrees of freedom) for each volume having the baseline image as the reference for motion correction and normalized mutual information as the cost function. It is worth noting that MCFLIRT employs a global-local hybrid optimization method for robust affine registration that is specifically tailored to brain images. Within a multiresolution framework, four scales were used (8, 4, 2, and 1 mm, i.e., supervoxel vs. subvoxel). At each scale, volumes were resampled after initial filtering to reduce the effect of noise. Further, we tested motion correction based on denoised HARDI sequences using the Joint Rician LMMSE filter (43) implemented as part of 3D Slicer (www.slicer.org), and found that the quantified motion with and without noise reduction was very similar.

**Figure 2:**
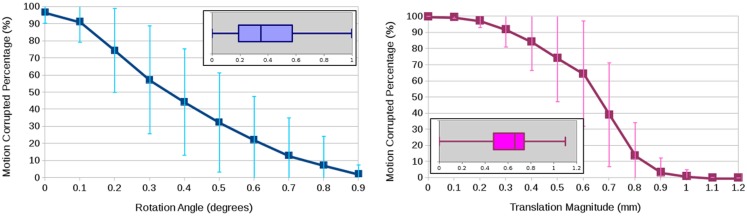
**Average and standard deviation of the percentage of motion-corrupted gradient directions as a function of thresholding on the estimated rotation angle in degrees (left) and the estimated translation magnitude in millimeter (right) for three human phantoms scanned twice at four clinical sites**. The boxplots show the overall statistics of estimated motion parameters.

To quantify motion, we used the magnitude of the translation vector (in millimeters) as well as the axis–angle rotation representation (in degrees) (4). The boxplots in Figure 2 show the rotational and translational components of the motion being detected from a total of 24 DWI datasets, showing an average of 0.39° rotation and 0.61 mm translation. The graphs in Figure 2 illustrate the arbitrariness of a common calculation of percentage of motion correction to determine the number of affected scans, here shown as a function of thresholding on the estimated motion parameters. While this experiment attributes the estimated rotation and translation parameters to actual subject motion, a part of the experimentally obtained parameters may be due to some imaging/image-processing uncertainty and also to image differences due to anatomical properties of the object (e.g., tissue orientation) that make the images “look” different even if they were perfectly aligned. To backup our analysis, we conducted another experiment where we contaminated a single DWI dataset with two independent realizations of Rician noise such that the two generated DWI images were perfectly aligned because they were the exact same image. Then, we ran motion correction where all DWI images were aligned to the same baseline, we obtain similar motion parameters although we are registering two independent acquisitions of the same subject. We therefore conclude that the transformation parameter estimates from FSL-MCFLIRT (31) are resilient to noise and may primarily caused by subject motion during a DWI scan, or eventually also by relative motion between subject and scans if considering artifacts due to pulse sequence and scanner technology.

### 2.2 Living phantom: Acquisition and gold standard generation

Unlike conventional MRI, where realistic phantoms exist (44), there is no widely acceptable realistic DWI phantom for the assessment of different processing tasks (43, 45). Existing phantoms simulate crossing sections in two and three dimensions, but they are not representative of white matter complex architecture with multiple fiber crossing, bending, and branching. The lack of realistic phantoms motivates us to base our analysis on living (human) phantoms being scanned under well-controlled environments and propose a HARDI-based QC to yield motion- and noise-free datasets. Acquired DWIs were preprocessed (refer to Figure 1A) to obtain nearly *noise-free* and *motion-free* datasets according to the following pipeline, and therefore, to be used as a *gold standard* for reconstruction and tractography.

#### 2.2.1 HARDI-based quality control

The QC process starts with identifying individual volumes having fast bulk (intra/within-gradient) motion using the signal dropout score proposed in Ref. (14). The score was computed for each slice in each volume, where slices with a score >1 were considered to have suspect signal dropout. Based on a zero-tolerance strategy, any volume having at least one slice with signal dropout was excluded from further analysis. It is worth noting that no within-gradient motion was detected in our phantom acquisitions. Each gradient was then independently denoised to reduce noise using the Rician LMMSE estimator with an 11 × 11 neighborhood (46) implemented in 3D Slicer (www.slicer.org) where the noise parameter is automatically estimated. Using DTIPrep (1), interslice brightness artifacts were detected via normalized correlation analysis between successive slices within a single DWI volume, where corrupted gradients were excluded before being streamed into the next steps. Further, interlaced correlation analysis (1) was used for detection and removal of Venetian blind artifacts (seen when motion occurs between the interleaved parts of an individual gradient volume) and fast bulk motion within a single DWI volume, where no quantitative within-gradient motion was detected.

For each DW-MRI scan, iterative FSL-MCFLIRT (20) was used to correct for intergradient subtle motion (<1° rotation and < 0.8 mm translation), with the baseline volume as the reference for rigid alignment (i.e., six degrees of freedom with normalized mutual information as the cost function). The corresponding diffusion-weighting gradient vectors were reoriented accordingly (37). To palliate the effect of spatial intensity inhomogeneities, N4 correction (47) was performed where the bias field was computed from the baseline volume and subsequently applied to all diffusion-weighted images. For further noise reduction, the Joint LMMSE (43) (www.slicer.org) was used to exploit the joint information from neighboring gradients from motion-corrected sequences. To avoid over-blurring, we used a 2 × 2 × 2 neighborhood with six neighboring gradients.

#### 2.2.2 Atlas-guided parcelation

For automated tractography selection and the quantification of whole brain connectivity, we defined a subject-specific unbiased atlas via DTI-derived data from HARDI sequences belonging to the same subject/phantom. This results in a tensor atlas, where we can define a detailed parcelation of neuroanatomical structures, and map it back to each raw scan. This reduces registration variability between each phantom data when defining the parcelation in subject spaces. The full process entails atlas creation and parcelation definition, as detailed in the following.

##### 2.2.2.1 Co-registration and atlas building

To define a common reference space, our framework is centered around the creation of a DTI atlas, generated as an unbiased average atlas from the study dataset via a deformable atlas building strategy. Unbiased atlas building is used to provide one-to-one mapping between the image data and the template atlas, wherein the atlas is built from the population of data as the centered image with the smallest deformation distances. The overall registration framework, similar to what has been presented in Ref. (48), proceeds in four steps: (1) image preprocessing via skull-stripping and tensor estimation, (2) affine alignment, (3) unbiased diffeomorphic atlas computation via GreedyAtlas module in AtlasWerks^3^ software (49), and (4) a refinement step via symmetric diffeomorphic registration using the advanced normalization tools – ANTS (32).

*Image preprocessing*. A brain masking is first performed on the baseline images using FSL-BET2 (brain extraction tool) (50) to remove all non-brain parts of the image. BET2 uses a surface model approach to robustly and accurately carry out the segmentation. We then model tensors using the brain masks from the initial DWI datasets by using weighted least squares estimation, and then extract related scalar maps such as fractional anisotropy (FA) images.

*Affine alignment*. The second step applies affine registration of baseline images to a previously defined baseline template. A multithreaded, coarse-to-fine registration scheme using mattes mutual information metric is employed in that regard (33). The transformations are applied to curvature FA maps. The use of curvature FA as feature to derive registration has initially been presented by Goodlett et al. (51). It is defined as the maximum eigenvalue of the Hessian of the FA image, therefore measuring image intensity curvature (second derivative) in the direction of largest curvature, which acts like a 3D ridge detector. It is computed by convolution of the FA image with a set of Gaussian second derivatives with a fixed aperture, proportional to the size of the white matter structures. The curvature feature image proved to be an efficient detector of the 3D manifold skeleton of major fiber bundles, which occur as tubular or sheet-like thin structures (similarly to the TBSS software), with the strongest response at their center. It is thus commonly used by our group when building population atlases to optimize correspondence of fiber tract geometries, and integrated into our freely distributed software package (48). The curvature FA maps are thus mapped to this template space, and then the intensity is rescaled via histogram matching.

*Atlas building*. We then use an unbiased deformable atlas-building procedure (52) that applies large deformation diffeomorphic metric mapping transformations to these intensity rescaled mapped curvature FA images. The procedure relates individual datasets to the subject-specific atlas template space by means of non-linear, invertible transformation. Tensor maps are transformed into the atlas space with tensor reorientation by the finite strain approach (53), taking into account both affine transformation and non-linear deformation. The transformed tensor images are finally averaged using the Riemannian framework proposed in Fletcher and Joshi (54), resulting in a three dimensional average tensor atlas.

*Atlas refinement*. An additional step is performed by direct symmetric diffeomorphic registration of initial FA images to the previously created DTI-FA atlas via the Advanced Normalization Tools – ANTS (32). In our experience, this dual stage procedure has been shown to produce a sharper atlas with improved registration accuracy, most likely attributable to the use of local normalized cross-correlation as the image similarity metric. Final affine transformation and deformation fields are then available from subject space to atlas space.

##### 2.2.2.2 White matter parcelation

We used the publicly available JHU-DTI-SS (a.k.a. “Eve”) atlas described in Oishi et al. (55). Defined as a single subject template, it includes both structural (T1w, T2w) and DTI images with white matter map parcelations, defining 176 hand-segmented core and peripheral regions of interest (ROIs). A multithreaded, coarse-to-fine diffeomorphic registration scheme using the cross-correlation metric via ANTS is employed on FA images between the Eve atlas and the subject-specific atlas. The computed deformation field is then applied to the Eve white matter label map. We can then map the parcelation, now defined in our subject atlas space, back to raw data in the initial image space, via the use of previously computed displacement fields. On a specific note, we concatenated the transformations from Eve atlas space to our initial images in order to directly map the parcelation and avoid the use of multiple interpolations. The white matter parcelation map is then defined both in the subject-specific atlas space and in each individual subject space.

### 2.3 Subject motion: Between simulation and correction

#### 2.3.1 Human motion simulation

As a pilot study, one human phantom was asked to be rescanned with his head tilted to simulate noticeable motion. The two datasets, after being QCed (see 1), were then used to construct motion-corrupted sequences (see Figure 1B). Based on the alignment of the baseline images of the two scans (original and tilted) using FSL-MCFLIRT, about 12° of rotation and 7 mm of translation were detected, whereas <1° of rotation and 0.8 mm of translation were found when aligning individual DWIs to their corresponding baseline image. It is worth noting that the quantified motion between the acquired datasets (i.e., untilted versus tilted brains) can be classified as severe subject motion (36). We then arbitrarily considered the first out of the two scans as the *“motion-free”* sequence and used it as a reference for performance evaluation of different motion correction schemes. A random percentage of DW images (10, 30, 50, 70, and 90%, each with five distinct random sets of gradient directions) drawn from the second scan (tilted brain) were mixed with the first scan to construct 25 motion-corrupted datasets. Noisy sequences were generated by simulating Rician noise based on seven levels of SNRs from 4 to 20 (56), yielding 175 (5 experiments × 5 corruption percentages × 7 SNR levels) sequences.

#### 2.3.2 Motion correction schemes

Correction for subject motion involves four main decision variables (see Figure 1C), where each distinct combination of choices defines a motion correction scheme. The first variable is which reference volume is to be used in the alignment process. Two options are available (20): *baseline-based* [e.g., Ref. (16)] and *model-based* [e.g., Ref. (35, 36)]. In this context, we use the FMAM (Fit Model to All Measurements) method (35) where target images for registration were generated by first fitting the diffusion tensor to the DWIs, followed by diffusion simulation to provide target images of similar contrast to the DWIs. Notice that with >50% motion corrupted, model-based reconstruction infers the spatial position/orientation from the gradients corresponding to the tilted brain due to its majority (i.e., gradients of the untilted brain are considered the motion-corrupted directions). Therefore, with model-based correction for sequences having more than 50% corrupted directions, the tilted brain was used as a reference for performance evaluation.

The second variable denotes whether the correction is performed based on *raw* or *denoised* DWIs, where the denoising process should not take into account joint information between diffusion gradients due to motion corruption. In our experiments, we denoised motion-corrupted sequences using the Rician LMMSE estimator (46), where each gradient was independently denoised.

The third variable entails the mode of correction, i.e., *registration-based* versus *outlier-based*. The first choice explores two options: (1) only aligning and interpolating the corrupted gradient directions to mimic the situation where a predefined motion parameter threshold is used to claim whether a DWI volume is motion-corrupted, (2) assuming there is always motion, forcing the alignment and interpolation of all DWI volumes. Note that both options involve the reorientation of the diffusion gradient vectors corresponding to the corrupted volumes (37) to incorporate the rotational component of subject motion. In the second choice, i.e., outlier-based, we mimic the motion scrubbing approach, where we exclude the affected gradient directions from subsequent computations (i.e., diffusion profile reconstruction and tractography). Eventually, the interpolation step in the registration-based choices introduces the fourth variable where we study the impact of using trilinear and sinc interpolants.

It is important to stress that, in our motion simulation paradigm (i.e., randomly mixing DW volumes from a tilted-brain dataset), the identity of the motion-corrupted directions is known *a priori* without any use of parameters. This prior information is used via the outlier-based correction, as well as the interpolate corrupted directions choices. Nonetheless, in practice, this *a priori* information corresponds to heuristically set thresholds on the estimated motion parameters beyond which volumes are claimed to be corrupted/outliers. For example, a rotation threshold of 0.5° and a translation threshold of about one voxel spacing are set by default in DTIprep (1).

### 2.4 Reconstruction and tractography

The reconstruction and whole brain tractography were computed for the motion corrected sequences as well as the motion-free sequences [gold standard generated in Section 2, followed by automatic tractography selection for seven major fiber bundles (see Figure 1D)].

We employed the constrained spherical deconvolution (CSD) technique (57) to reconstruct fiber orientation distributions functions (fODFs) from the DWI data using the diffusion imaging Python (DiPy) library (58). The fiber response function was estimated from the corpus callosum region, defined by the white matter parcelation (see 2), where it is known to have single fibers. In particular, we used an ROI at the center of the corpus callosum and of a radius that would include all its voxels. The response function was estimated in that region from the voxels with FA higher than 0.7.

Part of our analysis is based on comparing brain connectivity graphs, which are represented as weighted graphs and computed from fiber tractography results. Whole brain tractography was performed using the EuDX deterministic tracking technique (59), which is implemented in the DiPy library (58), using random seeding inside the brain region and a turning-angle threshold of 30° between two connected voxels [as suggested by Parizel et al. (60) to provide sufficient fiber density while minimizing the number of spurious tracts].

To extract brain connectivity graphs from the fiber tractography results, we used the 176 core and peripheral ROIs defined in the white matter parcelation (see 2). Let *N_ij_* denote the total number of streamlines connecting the *i*-th and *j*-th ROIs, each with length $l_{k}^{\mathrm{ij}}\forall k\in \left[1,N_{\mathrm{ij}}\right]$, and the edge weights *w_ij_* computed as follows (61): $w_{\mathrm{ij}}=\frac{1}{N_{\mathrm{ij}}}\sum_{k=1}^{N_{\mathrm{ij}}}\;\frac{1}{l_{k}^{\mathrm{ij}}}$. The normalization by the tracts length gives a higher connection strength to short tracts to compensate for the signal attenuation as a function of tract length. It is worth noting that the concept of using the connection strength or other measures to weight the graph edges was previously discussed in several papers [e.g., Ref. (62, 63)].

For tract-based analysis, an automatic tractography selection method was performed to select a subset of detected tracts from the whole brain tractography result corresponding to a specific white matter structure. Starting from the Eve-atlas-based white matter parcelation map defined in the subject space (see 2), the pass-through and not-pass-through volumes of seven fundamental fiber bundles (left and right hemispheres) were defined. To remove fibers that do not belong to the pathway of interest, we used the geometrical constraints specific for different fiber bundles as defined in Ref.(64), where the anatomical characteristics of these fiber bundles are defined in Ref. (65). We report the matching results from seven major fiber bundles: corpus callosum (CC), cingulum of the cingulate gyrus (CG), corticospinal tract (CST), fornix (FX), inferior fronto-occipital tract (IFO), inferior longitudinal fasciculus (ILF), and uncinate fasciculus (UNC).

### 2.5 Motion correction consequences: Evaluation metrics

The influence of various motion correction choices on subsequent reconstruction and tractography is evaluated according to voxel-based, global connectivity-based as well as tract-based metrics (see Figure 1E), detailed as follows.

#### 2.5.1 Voxel-based metrics

In order to measure similarities between the original motion-free fODFs and the fODFs corresponding to the motion corrected images, we use the Jensen–Shannon divergence (JSD), which has been used to quantify differences between ODFs in various studies, e.g., Ref. (66, 67). Given two probability distributions *P* and *Q*, the JSD metric is defined as follows:

(1) $$\mathrm{JSD}\left(P∥Q\right)=\frac{1}{2}\left[D_{\mathrm{KL}}\left(P∥M\right)+D_{\mathrm{KL}}\left(Q∥M\right)\right],$$

where *M* = (*P* + *Q*)/2 and *D_KL_* is the Kullback–Leibler divergence. In our case, *P* and *Q* are represented as discrete distributions; therefore, the KL divergence takes the following form: $D_{\mathrm{KL}}\left(P∥Q\right)=\sum_{i}\;P_{i}\log\frac{P_{i}}{Q_{i}}$, where *i* is the discrete sample index. The JSD is for PDFs, but we compute it for normalized fODFs. We believe that it is a good measure since it reveals subtle changes in PDFs so we can also keep track of changes in fiber volumes as well as orientations.

In addition to comparing fODFs, we are interested in quantifying local deviations in fiber orientations due to motion correction. Since brain connectivity maps are inferred by tracking local fiber orientations extracted from fODFs, distortions in those directions may lead to unreliable brain connectivity maps. Therefore, it is important to study the impact of motion correction on fiber orientations by directly comparing the local fiber orientations before and after correction. To that end, we use the mean angular deviation measure θ defined as follows:

(2) $$\theta_{i,j}^{k}=\frac{180}{\pi }\left|\cos^{-1}\left(v_{i}^{k}.v_{j}^{k}\right)\right|,\theta =\frac{1}{N}\sum_{k=1}^{N}\;\theta_{i,j}^{k},$$

where *N* is the number of fibers compared, and $v_{i}^{k}$ and $v_{j}^{k}$ correspond to the orientations of fiber *k*, with and without motion correction. Before averaging the deviations, we match the fibers, such that fiber *j* has the closest direction to fiber *i*. If the number of fibers is different, we compare the fibers that are present in both voxels. For example, if we have three fibers after motion correction, whereas before correction there were only two, we compare the two closest fiber directions. The fiber orientations were computed using the DiPy peak extraction tool (with 0.4 relative peak threshold and 20° minimum separation angle). We allowed up to five orientations in each voxel (*N* = 5). Since general image transformation does not necessarily preserve the original ordering of the fiber orientations, we first match the fibers based on the angular distance between each pair before computing the mean deviation.

#### 2.5.2 Global connectivity-based metric

Once the brain connectivity graphs were generated for the different sequences, we compared them by means of the graph diffusion distance (GDD) metric, which has been proposed in Ref. (68). The GDD is a novel distance measure for comparing weighted graphs, which takes into account the graph structure in addition to the edge weights, compared to the Frobenius norm, which is sensitive only to the edge weights. For an explanation of the differences between the GDD and the Frobenius norm, see the Barbell graph example in Ref. (68).

The GDD is based on the diffusion maps framework (69). Let *A*_1_ and *A*_2_ be weighted adjacency matrices for *N* vertices, that is, *A*_1_ and *A*_2_ are symmetric, non-negative, *N* × *N* real matrices with zeros along the principle diagonal. The (unnormalized) graph Laplacian operator is defined by *L_n_* = *D_n_* − *A_n_* (for *n* = 1, 2), where *D_n_* is a diagonal degree matrix for the adjacency *A_n_*, i.e., ${\left(D_{n}\right)}_{i,i}=\sum_{j=1}^{N}\;{\left(A_{n}\right)}_{i,j}$.

Given adjacency matrices *A*_1_ and *A*_2_, the columns of the Laplacian exponential kernels, exp(-*tL*_1_) and exp(-*tL*_2_), describe the different diffusion patterns centered at each vertex generated by diffusion up to time *t* under the two sets of weighted edges. Measuring the sum of squared differences between these patterns, summed over all the vertices, yields

(3) $$\begin{array}{llll} \xi_{\mathrm{gdd}}^{2}\left(A_{1},A_{2};t\right) & =\sum_{i,j}\;{\left({\left(\exp\left(-tL_{1}\right)\right)}_{i,j}-{\left(\exp\left(-tL_{2}\right)\right)}_{i,j}\right)}^{2}\; & & \;\\ & =||\exp\left(-tL_{1}\right)-\exp\left(-tL_{2}\right)|{|}_{F}^{2} \end{array}$$

where ||⋅||*_F_* is the matrix Frobenius norm. This defines a family of distance measures ξ, depending on the information propagation time *t*. The graph diffusion distance is given by ξ at the time of maximal difference, i.e., *d_gdd_*(*A*_1_, *A*_2_) = max*_t_* ξ*_gdd_*(*A*_1_, *A*_2_; *t*). Here, we compute *d_gdd_*(*A*_1_, *A*_2_) by first diagonalizing *L*_1_ and *L*_2_ and using the exponential mapping. Then, Equation (3) allows the computation of ξ(*A*_1_, *A*_2_; *t*) for any fixed *t*. Finally, we optimize over *t* by a line search to give *d_gdd_*(*A*_1_,*A*_2_).

#### 2.5.3 Tract-based metric

The spatial matching between motion-free and motion-corrected tracts was examined using Cohen’s Kappa statistic (70). The streamlines for a specific fiber tract (e.g., CST, IFO.) are first converted to a binary volume with the same dimension and spacing of the raw DWI, where voxels that were occupied by at least one streamline were assigned a value 1. The two tracking results to be matched were then superimposed to identify: (1) voxels that did not contain streamlines in either result (NN), (2) voxels that contain streamlines in both results (PP) and (3) voxel that contain streamlines in one of the results (PN or NP)^4^. The Kappa statistic measures the level of agreement of the tracking results and corrects for agreement expected by chance. Hence, Kappa is computed based on the probability of agreement *P*(*a*) and the probability of expected agreement due to chance *P*(*e*) as (71),

(4) $$\kappa =\frac{P\left(a\right)-P\left(e\right)}{1-P\left(e\right)},$$

where,

$$\begin{array}{llll} P\left(a\right) & =\frac{\mathrm{NN}+\mathrm{PP}}{\mathrm{PP}+\mathrm{PN}+\mathrm{NP}+\mathrm{NN}},\; & & \;\\ P\left(e\right) & =\frac{\left(\mathrm{NP}+\mathrm{PP}\right)\left(\mathrm{PN}+\mathrm{PP}\right)+\left(\mathrm{NP}+\mathrm{NN}\right)\left(\mathrm{PN}+\mathrm{NN}\right)}{{\left(\mathrm{PP}+\mathrm{PN}+\mathrm{NP}+\mathrm{NN}\right)}^{2}}.\; & & \; \end{array}$$

## 3 Results

The fODFs and the whole brain tractography were computed for the 3,150 motion corrected sequences (175 datasets × 18 correction schemes), as well as the motion-free sequences, followed by automatic tractography selection for seven major fiberbundles.

### 3.1 Voxel-based metrics

**Figure 3:**
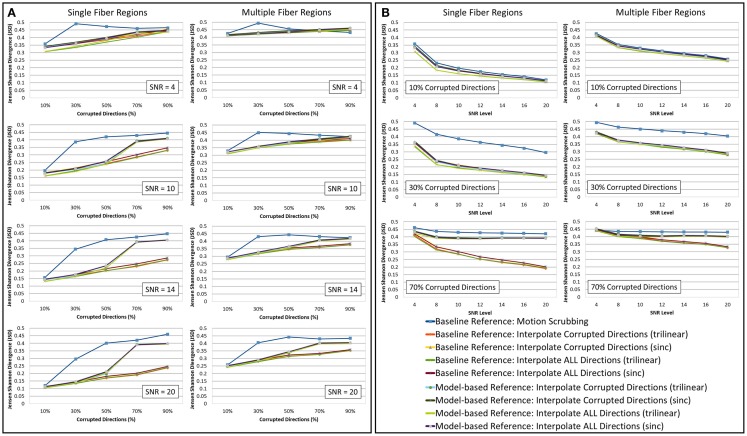
**The average Jensen–Shannon divergence (JSD) values (lower is better) for reconstructions based on raw datasets (denoised ones share similar performance) as (A) a function of motion corrupted percentage for different SNR levels and (B) a function of SNR levels for different motion corrupted percentage**. The first and third columns show JSDs single fiber regions while the second and fourth columns show such values for reconstructions based on multiple fiber regions. Notice the impact of motion scrubbing (removing gradient directions), which becomes more significant with more motion-corrupted directions when compared to registration-based correction. Further the impact of motion scrubbing is rendered evident for 10% corrupted gradients.

**Figure 4:**
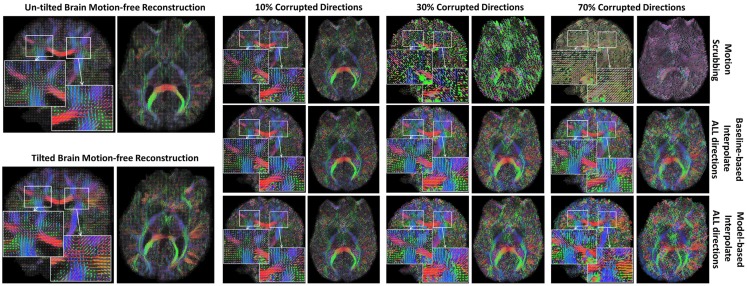
**Sample fODFs reconstruction from untilted and tilted motion-free datasets as well as reconstruction from motion-corrected datasets with 10, 30, and 70% corrupted gradient directions**. Correction choices shown include outlier-based (i.e., motion scrubbing) and registration-based (using baseline and model-based reference volumes).

**Figure 5:**
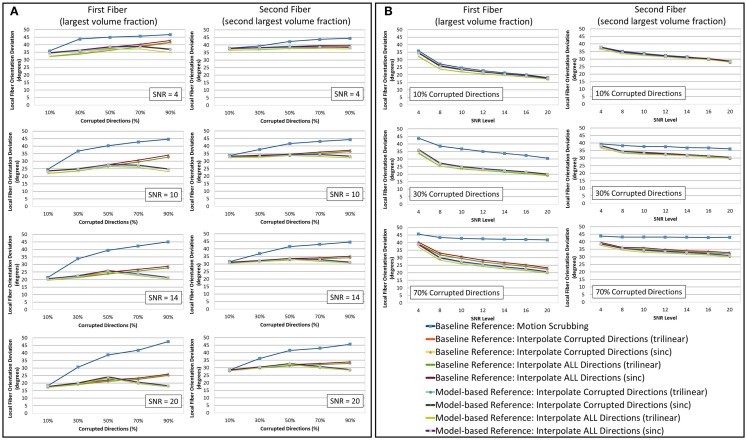
**The average fiber orientation deviation (lower is better) for reconstructions based on raw datasets (denoised ones share similar performance) as (A) a function of motion corrupted percentage for different SNR levels and (B) a function of SNR levels for different motion corrupted percentage**. The first and third columns show orientation deviation for the first detected fiber having the largest volume fraction while the second and fourth columns show such values for the second detected fiber having the second largest volume fraction. Notice that local fiber orientations are more affected by motion scrubbing as SNR decreases and/or corrupted directions increase.

The average JSD metric was computed using the fODF reconstruction from the “motion-free” dataset, not corrupted by mixing DWI directions from the tilted-brain scan, as a reference (i.e., presenting only subtle motion inherent to a scan). We differentiated between regions where multiple fibers were detected versus single fiber regions. Figure 3 shows the average JSD values for single and multiple fiber regions as a function of motion corrupted percentage for different SNR levels and as a function of SNR levels for different motion corrupted percentages. Figure 4 illustrates sample reconstructions from motion-free versus motion-corrected datasets for different corrupted percentages and different motion correction choices. Table 1 shows the effect of the denoising process prior to applying motion correction on the average JSD values for single and multiple fiber regions as a function of SNR levels for different motion corrupted percentages. Figure 5 shows the average deviation of local fiber orientations (for the first two dominant detected fibers per voxel) as a function of motion corrupted percentage, as well as SNR levels.

**Table 1:** **The effect of denoising on the average ± standard deviation of Jensen–Shannon divergence (JSD) values for single fiber regions and multiple fiber regions as a function of SNR levels for different motion corrupted percentages**.

Corrupted directions percentage	SNR levels						
**BASELINE-BASED MOTION CORRECTION (SINGLE FIBER REGIONS)**							
**30%**	**4**	**8**	**10**	**12**	**14**	**16**	**20**
Interpolate corrupted directions (trilinear): raw	0.360240 ± 0.045598	0.233751 ± 0.057661	0.206071 ± 0.056631	0.185168 ± 0.053253	0.168574 ± 0.050516	0.155135 ± 0.047892	0.135391 ± 0.043761
Interpolate ALL directions (trilinear): raw	0.334243 ± 0.059883	0.215623 ± 0.062502	0.194581 ± 0.060456	0.176716 ± 0.055924	0.162974 ± 0.052476	0.150333 ± 0.050609	**0.133550 ± 0.046793**
Interpolate corrupted directions (trilinear): denoised	0.352849 ± 0.040460	0.231980 ± 0.055656	0.202870 ± 0.054822	0.184626 ± 0.052840	0.167100 ± 0.049906	0.153834 ± 0.047516	0.135224 ± 0.043508
Interpolate ALL directions (trilinear): denoised	**0.329328 ± 0.059218**	**0.211255 ± 0.061444**	**0.190899 ± 0.058799**	**0.175393 ± 0.055475**	**0.161398 ± 0.051984**	**0.150292 ± 0.050625**	0.133699 ± 0.046593
**70%**	**4**	**8**	**10**	**12**	**14**	**16**	**20**
Interpolate corrupted directions (trilinear): raw	0.410600 ± 0.031331	0.318959 ± 0.050478	0.286541 ± 0.055425	0.252046 ± 0.057168	0.230605 ± 0.055139	0.214138 ± 0.053860	**0.190215 ± 0.048155**
Interpolate ALL directions (trilinear): raw	0.402799 ± 0.036878	0.314221 ± 0.054400	0.284747 ± 0.059198	0.252745 ± 0.059908	0.233456 ± 0.057853	0.216581 ± 0.056865	0.192958 ± 0.051800
Interpolate corrupted directions (trilinear): denoised	0.402564 ± 0.029625	0.313242 ± 0.049651	0.284339 ± 0.052764	**0.250802 ± 0.056173**	**0.231370 ± 0.054575**	**0.208578 ± 0.052739**	0.190779 ± 0.047920
Ine interpolate ALL directions (trilinear): denoised	**0.398054 ± 0.038274**	**0.310018 ± 0.054334**	**0.282856 ± 0.057399**	0.251609 ± 0.058948	0.234186 ± 0.057342	0.210697 ± 0.055260	0.194545 ± 0.051417
**BASELINE-BASED MOTION CORRECTION (MULTIPLE FIBER REGIONS)**							
**30%**	**4**	**8**	**10**	**12**	**14**	**16**	**20**
Interpolate corrupted directions (trilinear): raw	0.429747 ± 0.014377	0.374981 ± 0.028244	0.357056 ± 0.032396	0.335591 ± 0.035592	0.319182 ± 0.036475	0.304666 ± 0.037237	0.281099 ± 0.037361
Interpolate ALL directions (trilinear): raw	0.420579 ± 0.017062	0.365135 ± 0.029617	0.349272 ± 0.032369	0.330066 ± 0.034023	0.316609 ± 0.034180	0.300648 ± 0.035244	**0.278706 ± 0.034553**
Interpolate corrupted directions (trilinear): denoised	**0.408211 ± 0.013658**	0.361212 ± 0.027941	0.345386 ± 0.032180	0.328742 ± 0.035251	0.314137 ± 0.036364	0.300747 ± 0.036693	0.279468 ± 0.036236
Interpolate ALL directions (trilinear): denoised	0.415004 ± 0.016909	**0.357705 ± 0.029817**	**0.342500 ± 0.032743**	**0.325475 ± 0.033581**	**0.312227 ± 0.034740**	**0.300567 ± 0.034321**	0.279097 ± 0.033992
**70%**	**4**	**8**	**10**	**12**	**14**	**16**	**20**
Interpolate corrupted directions (trilinear): raw	0.441668 ± 0.009944	0.406974 ± 0.020601	0.394218 ± 0.025143	0.371914 ± 0.028719	0.359955 ± 0.030159	0.349868 ± 0.030453	0.326731 ± 0.028539
Interpolate ALL directions (trilinear): raw	0.438858 ± 0.010475	0.400544 ± 0.019045	0.387314 ± 0.024494	0.369097 ± 0.027007	0.358079 ± 0.028551	0.348511 ± 0.028087	0.327357 ± 0.026651
Interpolate corrupted directions (trilinear): denoised	**0.428357 ± 0.009559**	0.398647 ± 0.020752	0.387550 ± 0.024170	**0.364179 ± 0.028253**	0.353670 ± 0.029828	**0.342159 ± 0.029064**	**0.324294 ± 0.027985**
Interpolate ALL directions (trilinear): denoised	0.434734 ± 0.010609	**0.396470 ± 0.019646**	**0.385026 ± 0.023381**	0.364260 ± 0.026451	**0.353628 ± 0.027830**	0.343153 ± 0.026418	0.326491 ± 0.025983
**MODEL-BASED MOTION CORRECTION (SINGLE FIBER REGIONS)**							
**30%**	**4**	**8**	**10**	**12**	**14**	**16**	**20**
Interpolate corrupted directions (trilinear): raw	0.362824 ± 0.044584	0.234789 ± 0.057672	0.202436 ± 0.054992	0.185053 ± 0.052774	0.168921 ± 0.051011	0.154412 ± 0.048760	**0.137016 ± 0.044368**
Interpolate ALL directions (trilinear): raw	0.341529 ± 0.055268	0.216935 ± 0.061572	0.190353 ± 0.057184	0.177338 ± 0.053845	0.164345 ± 0.051940	**0.151763 ± 0.049245**	0.137129 ± 0.044860
Interpolate corrupted directions (trilinear): denoised	0.355832 ± 0.038952	0.233300 ± 0.055329	0.200942 ± 0.053886	0.183426 ± 0.051732	0.168669 ± 0.050471	0.156186 ± 0.048501	0.137568 ± 0.044271
Interpolate ALL directions (trilinear): denoised	**0.337800 ± 0.054166**	**0.214965 ± 0.060579**	**0.188046 ± 0.056021**	**0.174993 ± 0.053265**	**0.163170 ± 0.051443**	0.153666 ± 0.049561	0.139043 ± 0.045272
**70%**	**4**	**8**	**10**	**12**	**14**	**16**	**20**
Interpolate corrupted directions (trilinear): raw	0.437995 ± 0.020398	0.401102 ± 0.027875	0.395116 ± 0.029187	0.392917 ± 0.028751	0.394547 ± 0.029421	0.394157 ± 0.029765	0.393072 ± 0.029627
Interpolate ALL directions (trilinear): raw	0.424515 ± 0.023969	0.389511 ± 0.029832	0.385682 ± 0.030147	0.385935 ± 0.029025	0.389897 ± 0.030270	0.390043 ± 0.030282	0.389524 ± 0.030102
Interpolate corrupted directions (trilinear): denoised	0.433672 ± 0.019802	0.392704 ± 0.026322	0.385278 ± 0.027166	0.382104 ± 0.026334	0.382479 ± 0.027435	0.383047 ± 0.028099	0.382639 ± 0.027850
Interpolate ALL directions (trilinear): denoised	**0.423772 ± 0.023811**	**0.386144 ± 0.029342**	**0.380362 ± 0.029573**	**0.378202 ± 0.028394**	**0.380366 ± 0.029042**	**0.381810 ± 0.029858**	**0.382169 ± 0.029354**
**MODEL-BASED MOTION CORRECTION (MULTIPLE FIBER REGIONS)**							
**30%**	**4**	**8**	**10**	**12**	**14**	**16**	**20**
Interpolate corrupted directions (trilinear): raw	0.431485 ± 0.013919	0.374890 ± 0.027681	0.355161 ± 0.030889	0.340584 ± 0.034561	0.322469 ± 0.035754	0.306188 ± 0.036843	0.282984 ± 0.037859
Interpolate ALL directions (trilinear): raw	0.424731 ± 0.016649	0.366541 ± 0.029399	0.348776 ± 0.031170	0.336014 ± 0.033028	0.319378 ± 0.034755	0.303846 ± 0.035464	0.283342 ± 0.035411
Interpolate corrupted directions (trilinear): denoised	**0.409946 ± 0.012715**	0.361122 ± 0.027406	0.344173 ± 0.030754	0.331062 ± 0.034374	0.316224 ± 0.035922	0.302532 ± 0.036504	**0.281484 ± 0.036670**
Interpolate ALL directions (trilinear): denoised	0.420245 ± 0.016382	**0.360538 ± 0.029426**	**0.342159 ± 0.030712**	**0.328595 ± 0.032733**	**0.314057 ± 0.034207**	**0.300968 ± 0.035322**	0.283460 ± 0.034162
**70%**	**4**	**8**	**10**	**12**	**14**	**16**	**20**
Interpolate corrupted directions (trilinear): raw	0.452994 ± 0.010220	0.417146 ± 0.015161	0.410814 ± 0.016068	0.406033 ± 0.016404	0.408391 ± 0.017131	0.407360 ± 0.017038	0.402509 ± 0.019258
Interpolate ALL directions (trilinear): raw	0.441239 ± 0.011757	0.402898 ± 0.020790	0.398061 ± 0.020777	0.397453 ± 0.019135	0.401401 ± 0.018495	0.401485 ± 0.017602	0.395939 ± 0.018965
Interpolate corrupted directions (trilinear): denoised	0.448519 ± 0.009997	0.407138 ± 0.014850	0.399052 ± 0.015820	0.393196 ± 0.015699	0.393496 ± 0.016721	0.393950 ± 0.016122	0.389538 ± 0.017035
Interpolate ALL directions (trilinear): denoised	**0.440633 ± 0.011008**	**0.399459 ± 0.020710**	**0.390507 ± 0.021431**	**0.385709 ± 0.020485**	**0.387375 ± 0.020483**	**0.389474 ± 0.019294**	**0.384891 ± 0.019050**

*Bold indicates the motion correction scenarios with minimal effect on the JSD metric in case of denoised datasets*.

### 3.2 Global connectivity metric

**Figure 6:**
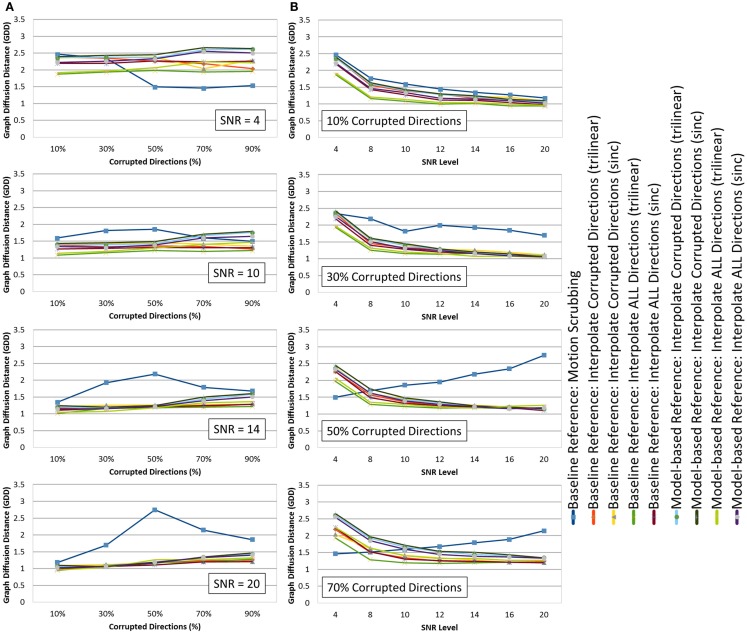
**The average graph diffusion distance (GDD) (lower is better) for the whole brain tractography derived from the raw datasets (denoised ones share similar performance) as (A) a function of the corrupted directions percentage for different SNR levels and (B) a function of SNR levels for different motion corrupted percentages**. Notice the different behavior displayed by motion scrubbing for ≥50% corrupted directions, which due to having more short tracts connecting nearby region of interests while being assigned to larger weights in the graph construction step.

**Figure 7:**
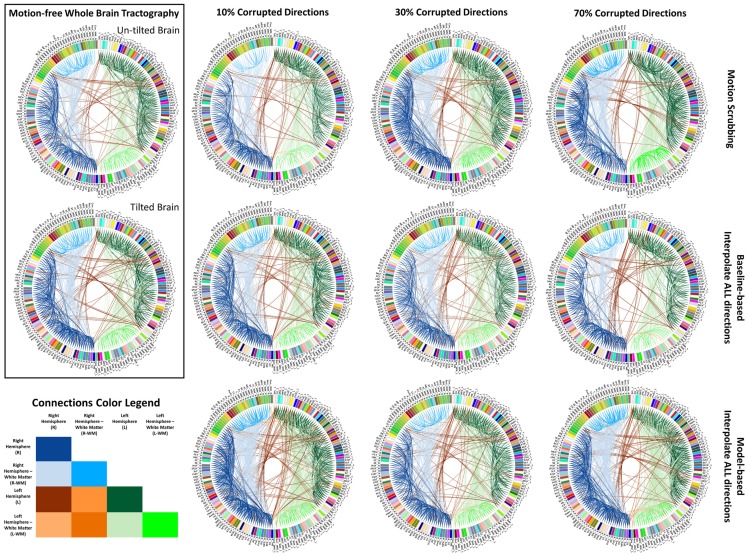
**Sample reconstructed connectomic profile (i.e., connectogram) from untilted and tilted motion-free datasets as well as connectograms from motion-corrected datasets with 10, 30, and 70% corrupted gradient directions**. Correction choices shown include outlier-based (i.e., motion scrubbing) and registration-based (using baseline and model-based reference volumes). Notice the tendency of motion scrubbing to add more links between nearby ROIs at corruption percentages, implying the detection of more short tracts.

Figure 6 shows the average graph diffusion distance (GDD) metric as function of both the corrupted directions percentage and the SNR levels. The metric compares the weighted connectivity graphs from the whole brain tractography of the “motion-free” dataset to that of the motion-corrected datasets. It is worth noting that the tractography of the tilted brain dataset is used as a reference for model-based corrections when the corrupted percentage exceeds 50%. Figure 7 visualizes the brain connectivity being represented circularly using the Circos software (72) where the parcelated structures (refer to Table S1 in Supplementary Material for their full names) are displayed on a connectogram representing left and right hemispheres symmetrically positioned along the vertical axis. The weighted connectivity matrix computed as described in Section 4 was normalized to attain a unit maximum. Each entry in the normalized connectivity matrix corresponds to an interregion link with thickness proportional to the entry weight. To avoid dense visualization, all entries with weight <0.15 were discarded.

### 3.3 Tract-based metric

**Figure 8:**
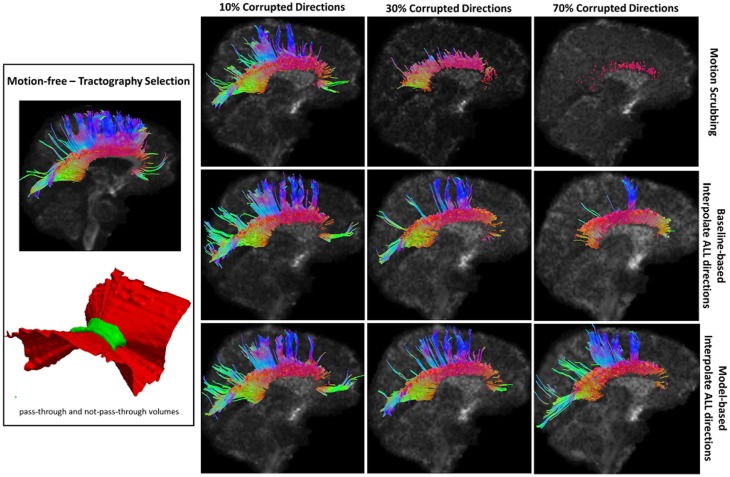
**Sample tractography selection for the corpus callosum (CC) from the untilted motion-free dataset as well as selections from motion-corrected datasets with 10, 30, and 70% corrupted gradient directions**. Correction choices shown include outlier-based (i.e., motion scrubbing) and registration-based (using baseline and model-based reference volumes). One can observe the short tracts being detected by motion scrubbing at high corruption percentages due to the exclusion of too many gradient directions.

**Figure 9:**
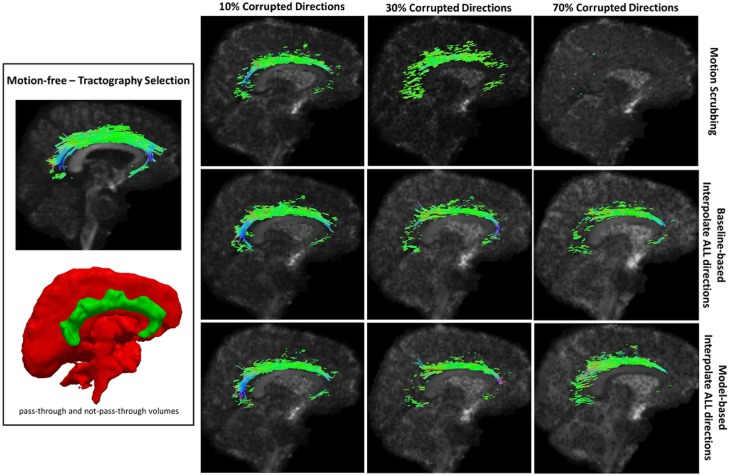
**Sample tractography selection for the cingulum of the cingulate gyrus (CG) from the untilted motion-free dataset as well as selections from motion-corrected datasets with 10, 30, and 70% corrupted gradient directions**. Correction choices shown include outlier-based (i.e., motion scrubbing) and registration-based (using baseline and model-based reference volumes). Notice the inability of motion scrubbing to detect an anatomically realized CG at high corrupted percentages.

**Figure 10:**
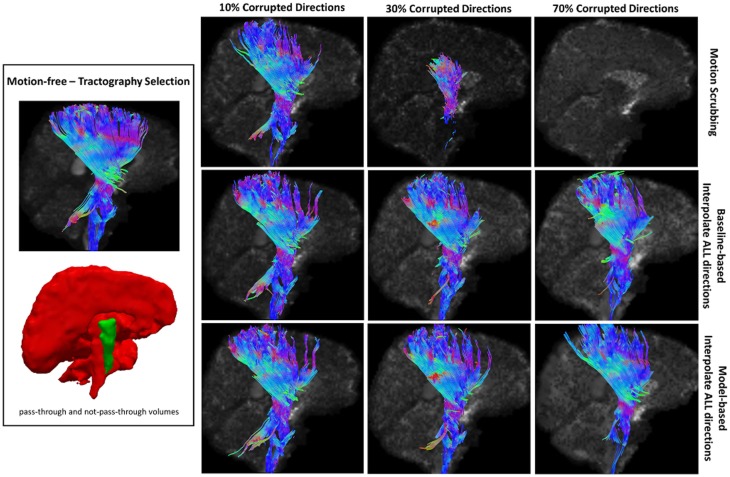
**Sample tractography selection for the corticospinal tract (CST) from the untilted motion-free dataset as well as selections from motion-corrected datasets with 10, 30, and 70% corrupted gradient directions**. Correction choices shown include outlier-based (i.e., motion scrubbing) and registration-based (using baseline and model-based reference volumes). Note that motion scrubbing cannot recover long tracts such as CST beyond 10% motion corruption.

**Figure 11:**
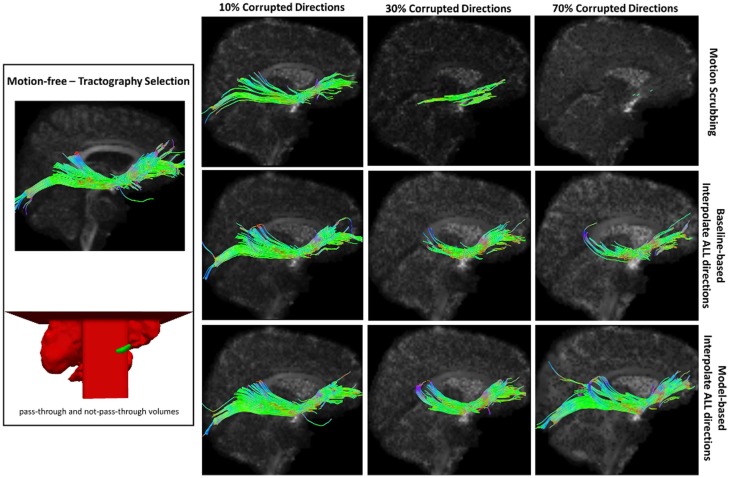
**Sample tractography selection for the inferior fronto-occipital tract (IFO) from the untilted motion-free dataset as well as selections from motion-corrected datasets with 10, 30, and 70% corrupted gradient directions**. Correction choices shown include outlier-based (i.e., motion scrubbing) and registration-based (using baseline and model-based reference volumes). Note that motion scrubbing cannot recover long tracts such as IFO beyond 10% motion corruption. Further, motion-based motion correction tends to recover longer tracts at high motion corruption compared to baseline-based correction.

**Figure 12:**
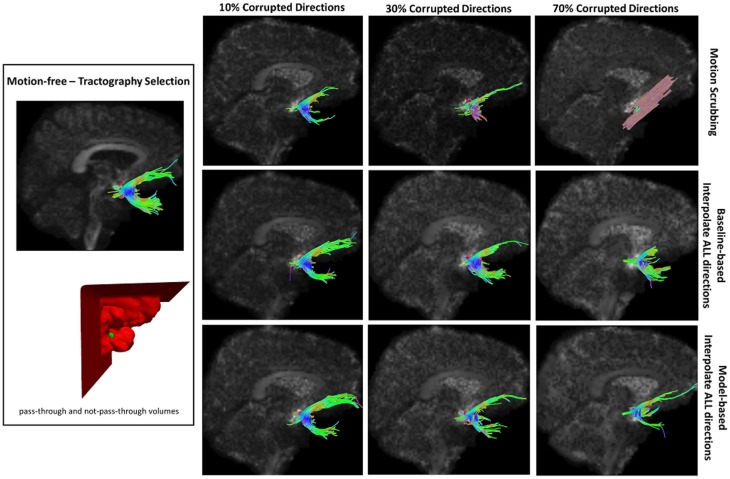
**Sample tractography selection for the uncinate fasciculus (UNC) from the untilted motion-free dataset as well as selections from motion-corrected datasets with 10, 30, and 70% corrupted gradient directions**. Correction choices shown include outlier-based (i.e., motion scrubbing) and registration-based (using baseline and model-based reference volumes). Notice the inaccurate UNC tract being detected from the motion scrubbing choice at high percentages of motion corruption.

Table 2 shows the average Cohen’s Kappa statistic computed for corpus callosum (CC), corticospinal tract (CST), and inferior fronto-occipital tract (IFO) (where other pathways showed similar trend) based on automatic tractography selection using whole brain tractography of raw datasets (denoised datasets showed similar trends due to the robust fODF estimation, yet their graphs were omitted due to space limitation). Figures 8–12 show sample tractography selections for the aforementioned tracts from the untilted motion-free dataset as well as selections from motion-corrected datasets with different corrupted gradient directions. Correction choices shown include outlier-based (i.e., motion scrubbing) and registration-based (using baseline and model-based reference volumes). Also pass-through (in green) and not-pass-through (in red) volumes (i.e., isosurfaces) are shown. Their definitions along with the geometric constraints employed to remove fibers, which do not belong to the pathway of interest, can be found in Ref. (64).

**Table 2:** **The average Cohen’s Kappa statistic (higher is better) of different anatomically defined fiber pathways (other pathways show similar trend) based on automatic tractography selection based on whole brain tractography of raw datasets (denoised ones share similar performance) for different corrupted directions percentages**.

	SNR levels						
**CORPUS CALLOSUM (CC)**							
**10%**	**4**	**8**	**10**	**12**	**14**	**16**	**20**
Baseline reference: motion scrubbing	0.337569	0.512323	0.549884	0.583176	0.608084	0.623132	**0.653595**
Baseline reference: interpolate corrupted directions (trilinear)	0.371873	0.527897	0.560684	0.597392	0.610865	0.625269	0.641443
Baseline reference: interpolate ALL directions (trilinear)	0.430934	**0.565719**	**0.604035**	0.612666	0.623576	**0.645637**	0.650286
Model-based reference: interpolate corrupted directions (trilinear)	0.372998	0.533756	0.56661	0.597997	0.610078	0.625306	0.645364
Model-based reference: interpolate ALL directions (trilinear)	**0.432495**	0.56421	0.590059	**0.616106**	**0.628666**	0.643482	0.648159
**30%**	**4**	**8**	**10**	**12**	**14**	**16**	**20**
Baseline reference: motion scrubbing	0.121185	0.240279	0.295196	0.342217	0.367067	0.397206	0.426858
Baseline reference: interpolate corrupted directions (trilinear)	0.344391	0.480168	0.510159	0.517193	0.519918	0.529172	0.536126
Baseline reference: interpolate ALL directions (trilinear)	**0.397277**	0.508548	0.520689	0.522688	0.528747	0.52865	0.531048
Model-based reference: interpolate corrupted directions (trilinear)	0.34037	0.483498	0.511051	0.522758	0.53595	0.536208	0.54493
Model-based reference: interpolate ALL directions (trilinear)	0.391228	**0.510303**	**0.522294**	**0.531674**	**0.541776**	**0.538634**	**0.546074**
**50%**	**4**	**8**	**10**	**12**	**14**	**16**	**20**
Baseline reference: motion scrubbing	0.195245	0.234216	0.24072	0.239568	0.234356	0.228969	0.212593
Baseline reference: interpolate corrupted directions (trilinear)	0.32114	0.43416	0.463943	0.456179	0.456507	0.455066	0.441334
Baseline reference: interpolate ALL directions (trilinear)	**0.354402**	**0.447591**	**0.476502**	**0.460336**	**0.464282**	0.455952	0.435115
Model-based reference: interpolate corrupted directions (trilinear)	0.308208	0.424219	0.454309	0.455936	0.456871	0.465054	**0.477503**
Model-based reference: interpolate ALL directions (trilinear)	0.344133	0.443797	0.459322	0.459972	0.462699	**0.46546**	0.47007
**70%**	**4**	**8**	**10**	**12**	**14**	**16**	**20**
Baseline reference: motion scrubbing	0.178267	0.178831	0.178042	0.163248	0.164247	0.158891	0.152246
Baseline reference: interpolate corrupted directions (trilinear)	0.314508	0.391142	0.408141	0.417553	0.420105	0.412033	0.405891
Baseline reference: interpolate ALL directions (trilinear)	0.327764	0.395117	0.405833	0.415301	0.421643	0.402026	0.401629
Model-based reference: interpolate corrupted directions (trilinear)	0.290382	0.405799	0.440177	0.452235	0.479685	**0.479166**	**0.504993**
Model-based reference: interpolate ALL directions (trilinear)	**0.330574**	**0.428458**	**0.455009**	**0.46612**	**0.482178**	0.478169	0.496215
**CORTICOSPINAL TRACT (CST)**							
**10%**	**4**	**8**	**10**	**12**	**14**	**16**	**20**
Baseline reference: motion scrubbing	0.255609	0.511582	0.59193	0.647802	0.681451	0.708302	0.733556
Baseline reference: interpolate corrupted directions (trilinear)	0.288567	0.568014	0.636962	0.674537	0.700027	0.714401	0.741004
Baseline reference: interpolate ALL directions (trilinear)	**0.383782**	**0.673544**	**0.709478**	**0.732921**	**0.735943**	0.74033	**0.753895**
Model-based reference: interpolate corrupted directions (trilinear)	0.290589	0.561405	0.636853	0.673316	0.699448	0.713441	0.739494
Model-based reference: interpolate ALL directions (trilinear)	0.377735	0.663838	0.703892	0.723948	0.732407	**0.741558**	0.751347
**30%**	**4**	**8**	**10**	**12**	**14**	**16**	**20**
Baseline reference: motion scrubbing	0.08445	0.181213	0.223927	0.250415	0.278556	0.304034	0.338546
Baseline reference: interpolate corrupted directions (trilinear)	0.282041	0.52623	0.598869	0.626852	0.636781	0.643479	0.651485
Baseline reference: interpolate ALL directions (trilinear)	**0.366403**	**0.61723**	**0.665015**	0.67397	0.663454	0.668005	0.671065
Model-based reference: interpolate corrupted directions (trilinear)	0.273331	0.521025	0.603001	0.639991	0.658976	0.671995	0.67978
Model-based reference: interpolate ALL directions (trilinear)	0.347568	0.612903	0.655906	**0.678086**	**0.6801**	**0.692068**	**0.686448**
**50%**	**4**	**8**	**10**	**12**	**14**	**16**	**20**
Baseline reference: motion scrubbing	0.167928	0.215559	0.227706	0.231256	0.238757	0.233776	0.237475
Baseline reference: interpolate corrupted directions (trilinear)	0.274988	0.47285	0.528849	0.56997	0.578886	0.581467	0.58602
Baseline reference: interpolate ALL directions (trilinear)	**0.329036**	**0.540045**	**0.567838**	**0.60573**	**0.596126**	0.594135	0.599463
Model-based reference: interpolate corrupted directions (trilinear)	0.25215	0.466852	0.518179	0.553814	0.56339	0.584337	0.596757
Model-based reference: interpolate ALL directions (trilinear)	0.300971	0.519456	0.551479	0.574403	0.578095	**0.603384**	**0.609796**
**70%**	**4**	**8**	**10**	**12**	**14**	**16**	**20**
Baseline reference: motion scrubbing	0.209264	0.213673	0.214178	0.214527	0.219481	0.206522	0.210003
Baseline reference: interpolate corrupted directions (trilinear)	0.268415	0.449839	0.495255	0.54836	0.560483	0.565741	0.553228
Baseline reference: interpolate ALL directions (trilinear)	**0.30485**	0.486243	0.531024	0.563493	0.569974	0.578043	0.561712
Model-based reference: interpolate corrupted directions (trilinear)	0.237249	0.43595	0.511829	0.537773	0.579497	0.591882	0.617357
Model-based reference: interpolate ALL directions (trilinear)	0.304681	**0.493711**	**0.554253**	**0.575569**	**0.605093**	**0.610975**	**0.62865**
**INFERIOR FRONTO-OCCIPITAL TRACT (IFO)**							
**10%**	**4**	**8**	**10**	**12**	**14**	**16**	**20**
Baseline reference: motion scrubbing	0.021174	0.164388	0.253179	0.360941	0.432971	0.522092	0.538292
Baseline reference: interpolate corrupted directions (trilinear)	0.036125	0.248734	0.350268	0.41586	0.467664	0.496367	0.525266
Baseline reference: interpolate ALL directions (trilinear)	**0.066164**	**0.404309**	0.453207	**0.49633**	**0.497915**	**0.542272**	**0.55883**
Model-based reference: interpolate corrupted directions (trilinear)	0.036877	0.241989	0.355478	0.41203	0.450946	0.497335	0.530589
Model-based reference: interpolate ALL directions (trilinear)	0.061719	0.397744	**0.476713**	0.481677	0.484301	0.532199	0.553205
**30%**	**4**	**8**	**10**	**12**	**14**	**16**	**20**
Baseline reference: motion scrubbing	0.017605	0.032846	0.036941	0.054356	0.069675	0.096068	0.120592
Baseline reference: interpolate corrupted directions (trilinear)	0.021015	0.193547	0.30352	0.358774	0.391792	0.417395	0.448851
Baseline reference: interpolate ALL directions (trilinear)	**0.040243**	**0.286059**	**0.375406**	0.407854	0.415109	**0.449185**	0.45364
Model-based reference: interpolate corrupted directions (trilinear)	0.019676	0.190149	0.298156	0.374981	0.394936	0.440116	0.450691
Model-based reference: interpolate ALL directions (trilinear)	0.036533	0.269425	0.356155	**0.41894**	**0.417197**	0.448939	**0.457867**
**50%**	**4**	**8**	**10**	**12**	**14**	**16**	**20**
Baseline reference: motion scrubbing	0.079802	0.096081	0.088847	0.08342	0.084004	0.078977	0.074762
Baseline reference: interpolate corrupted directions (trilinear)	0.023615	0.173538	0.255646	0.303376	0.355478	0.370352	**0.389862**
Baseline reference: interpolate ALL directions (trilinear)	**0.037031**	**0.21137**	**0.287878**	**0.332891**	**0.361154**	**0.383244**	0.388926
Model-based reference: interpolate corrupted directions (trilinear)	0.017528	0.155743	0.226579	0.260404	0.306267	0.343361	0.363517
Model-based reference: interpolate ALL directions (trilinear)	0.031326	0.187443	0.250407	0.282972	0.310855	0.353314	0.350486
**70%**	**4**	**8**	**10**	**12**	**14**	**16**	**20**
Baseline reference: motion scrubbing	0.103989	0.111274	0.105098	0.103238	0.107333	0.11481	0.110321
Baseline reference: interpolate corrupted directions (trilinear)	0.02605	0.137873	0.203676	0.292856	0.312459	0.329589	0.36963
Baseline reference: interpolate ALL directions (trilinear)	0.034815	0.169664	0.252561	0.300767	0.314673	0.347346	0.371886
Model-based reference: interpolate corrupted directions (trilinear)	0.021983	0.185256	0.299656	0.364072	0.433785	0.458342	0.477853
Model-based reference: interpolate ALL directions (trilinear)	**0.043441**	**0.249726**	**0.354267**	**0.409827**	**0.471097**	**0.479884**	**0.485465**

*Bold indicates the motion correction scenarios which yield maximal agreement of different fiber pathways to those obtained from motion-free sequence*.

## 4 Discussion

In this section, we discuss the impact of different motion correction choices using local as well as global metrics.

### 4.1 Voxel-based results

Heterogeneous regions are more affected by motion correction, showing larger average JSD in general when compared to the single fiber regions, regardless of the correction mode, interpolation scheme, or reference volume employed (see Figure 3).

The impact of motion scrubbing (removing gradient directions) becomes more pronounced with more motion-corrupted directions when compared to registration-based correction (see Figure 3A). Meanwhile, the JSD values indicate minimal deformations in fODFs reconstructed for baseline-based correction at high SNR levels compared to model-based correction, whereas both choices show comparable average JSD values at low SNR levels. This complies with the conclusions presented in Ref. (20).

Forcing the correction and interpolation of all gradient directions shows comparable performance compared to the correction and interpolation of only the corrupted directions (see Figure 3A). This observation discourages the choice of heuristic parameters on motion parameters beyond which directions are claimed to be corrupted and interpolated. Further, interpolation of all directions causes less impact on the reconstructed fODFs at low corrupted percentages (<50%). We can assume, therefore, that motion is omnipresent and can be corrected for by the alignment and interpolation of all gradient directions.

On the interpolation aspect of correction, the sampling theory suggests the sinc kernel as the ideal interpolation kernel; nonetheless, this gives rise to the Gibbs phenomena (i.e., ringing) due to kernel truncation. This explains the smaller fODF deformation when using trilinear interpolation compared to sinc interpolation. Trilinear interpolation, which is much faster, is probably sufficient for motion correction.

In Figure 3B, one can observe the comparable impact of different motion correction choices at low motion corruption percentages (<30%). Whereas with higher motion corruption, a situation that is encountered in studies including infants, for example, motion scrubbing shows a significant impact on the reconstructed fODFs even at high SNR levels. This effect is more pronounced in regions with crossing fibers where the ability to resolve fiber crossings is deteriorated especially as the separation angle of the fibers decreases.

Further, baseline-based motion corrections show minimal JSD values with higher corruption levels (> 50%) when compared to model-based corrections, regardless of the interpolation scheme employed. The difference in performance between baseline-based and model-based becomes more significant as the SNR level increases.

The denoising process yields smaller JSD values for low SNR levels (<12) (see Table 1), while providing comparable performance for baseline-based and model-based motion correction choices. The slight decrease of JSD values for denoised datasets compared to the raw ones is due to the fODF reconstruction processes where we use the constrained spherical deconvolution (CSD) technique (57). In an iterative manner, the deconvolution process in CSD applies a non-negativity constraint on the estimated fODFs as negative fiber orientation densities are physically impossible. This process provides fODFs estimates that preserve the angular resolution while being robust to noise. Yet, as a word of caution, the denoising process, when applied to motion-corrupted datasets, should not take into consideration the joint information from diffusion gradients since voxel-wise correspondence between different diffusion volumes is not guaranteed.

In Figure 4, one can observe the significant impact of motion scrubbing (i.e., outlier-based correction) on the reconstructed fODFs for mildly corrupted datasets (e.g., 30% corrupted directions). Further, it can be noticed that with >50% motion corruption, model-based reconstruction infers the spatial position from the gradients corresponding to the tilted brain due to its majority (i.e., gradients of the untilted brain are considered the motion-corrupted directions).

Due to the insufficient number of gradients and unbalanced sampling of the q-space, the impact of motion scrubbing on the estimated fiber orientations becomes evident as SNR decreases and/or corrupted directions increase (see Figure 5).

Although interpolating all directions versus corrupted directions reports comparable orientation deviation with lower impact on fractionally corrupted datasets (<50%), we still favor forcing such a process to all directions to avoid the *ad hoc* process of thresholding motion parameters.

Nonetheless, one can notice the peaked performance of the orientation deviation at 50% corrupted directions for model-based motion correction choices. The explanation of this phenomenon is based on the fact that, with >50% of the gradients being corrupted (i.e., corresponding to the tilted brain), the formed reference volumes would instead infer its anatomical structure from the tilted brain. For highly corrupted datasets, the gradients corresponding to the untilted brains become the corrupted directions (i.e., a 70% corruption will have a performance similar to the 30% case).

Model-based corrections display higher impact on the JSD of the reconstructed fODFs at higher levels of motion corruption, but such corrections have a smaller impact on the fiber orientation deviations especially when interpolating all directions (trilinear interpolant). This change of JSD metric implies an increase in the overall fODF volume when compared to the reconstructions from the *motion-free* dataset, yet the fODFs maintain the voxel-wise fiber crossing structure. This observation is more pronounced for fibers with the largest fiber volume fraction.

### 4.2 Global connectivity-based results

Whereas there is a slight performance difference between GDD values computed based on raw datasets versus those from denoised dataset, thanks to the fODF reconstruction that is robust to noise contamination, one may observe consistent findings when GDD is compared to the JSD metric. In particular, the global brain connectivity is least affected by the motion correction step when forcing the alignment and interpolation of all gradient directions without setting a predefined threshold to claim corrupted volumes. There is a significant difference between GDD values obtained from trilinear interpolation compared to sinc interpolation. This implies that the impact of sinc interpolation on the fODFs, being encoded by the JSD metric, yields global brain connectivity that is different from the “motion-free”-based brain connectivity.

Whereas the effect of motion correction is evident at higher corrupted percentages (except for motion scrubbing), one can notice the effect of noise where the impact of motion correction becomes more significant at low SNRs (<12), while different correction choices (except motion scrubbing) render slight performance difference at high SNRs (>12). Moreover, being consistent with different SNR levels, the baseline-based correction choices yield connectivity graphs with minimal deviations (smaller GDD) compared to their corresponding model-based choices.

On the contrary, motion scrubbing displays a different behavior. The GDD values from the scrubbed datasets, though maximal compared to the other correction choices, are decreasing with higher SNR levels for <50% corrupted directions, but this behavior is soon changed to the opposition direction for ≥50% corrupted directions (see Figure 6B). This change of behavior is perceivable in Figure 6A where the GDD values are maximal at 50% corruption percentage for high SNR levels (>12), whereas such a peak occurs even at low corrupted percentages (e.g., 30%) for low SNRs (<12). This phenomenon can be explained as follows: with high percentage of motion-contaminated gradients, the scrubbing (outlier-based) option tends to produce an inadequate set of gradients for accurate fODF estimation due to the exclusion of too many gradients. This unbalanced sampling of the q-space, henceforth, biases the CSD process to converge to an incorrect solution, producing inaccurate fiber orientation, and in turn imprecise brain connectivity. Hence, the increase of the GDD values with higher SNRs beyond 30% corrupted directions is due to having more short tracts connecting nearby region of interests while being assigned to larger weights in the graph construction step (see 4).

In Figure 7, one can observe the motion scrubbing behavior where the links become denser with higher corrupted percentages, implying the detection of more short tracts connecting nearby ROIs. On the other hand, the baseline-based choice reveals comparable connectograms to the motion-free ones while model-based counterpart tends to add more shorter tracts.

### 4.3 Tract-based results

Being consistent with the results from the other metrics, motion scrubbing shows a significant decrease in the degree of tract agreement when increasing the percentage of motion corruption, which in turn leads to discarding more gradient directions. With <50% corrupted directions, the tract agreement degree increases with higher SNR levels, yet such a trend changes with ≥50% where shorter or no tracts being detected, which deviates from being anatomically realistic; see, for example, the top row of Figures 8–12 where tracts can be even missed even at 70% corruption. The CST and IFO tracts are good examples of long tracts that are not recovered by motion scrubbing beyond 10% motion corruption, see Figures 10 and 11. Nonetheless, the maximal agreement is achieved when aligning and interpolating all gradient directions to correct for motion regardless of the reference volume used in the registration process (i.e., baseline versus model-based). It can be observed in Figures 8–12 that model-based motion correction is able to recover longer tracts at high corruption percentages compared to the baseline-based motion correction.

## 5 Conclusion: Guidelines for Motion Correction in HARDI Acquisitions

Although there is excellent theoretical work on DWI acquisition parameters and ODF reconstruction schemes, as well as their effects on the quality and crossing fiber resolution, standard users lack clear guidelines and recommendations on the best ways to approach and correct for motion in practical settings. This work investigated motion correction using transformation and interpolation of affected DWI directions versus the exclusion of subsets of DWIs, and its impact on the reconstructed fODFs, local fiber orientations, brain connectivity, and detection of fiber tracts. The various effects were systematically explored and illustrated via living phantom data, leading to the general conclusion that motion, even subtle, exists in every acquired DW scan and special care is needed to correct for motion. In the following, we summarize the findings of our analysis, which might serve as guidelines for users in practice:

– Although least recommended, motion scrubbing (removing corrupted gradient directions) can be used in studies with well-controlled environments and involving not-in-pain adults or sedated subjects, where minimal subject motion is anticipated (i.e., <10%motion corruption). Yet, this gradient removal should not result in unbalanced sampling of the q-space since the gradient distribution should be as uniform as possible on the sphere.
– Voxel-wise reconstructions, tractography, and global brain connectivity are least affected by the motion correction step when forcing the alignment and interpolation of all gradient directions without setting predefined thresholds to claim corrupted volumes.
– Using voxel-wise reconstructions that are robust to noise, the denoising process can be considered unnecessary prior to applying motion correction. Nonetheless, if applied, the denoising algorithms should not take into account joint information from different diffusion gradients since voxel-wise correspondence is not guaranteed.
– Baseline-based correction choices can be used in studies involving voxel-wise scalars, which depend on the volume of the reconstructed ODFs, especially with highly motion-corrupted datasets.
– Model-based correction choices, on the other hand, are recommended for studies requiring the recovery and analysis of long tracts, e.g., CST and IFO, especially with highly motion-corrupted datasets.
– Trilinear interpolation, although much faster compared to sinc, is probably sufficient for motion correction, where the global brain connectivity is least affected.

**Figure 13:**
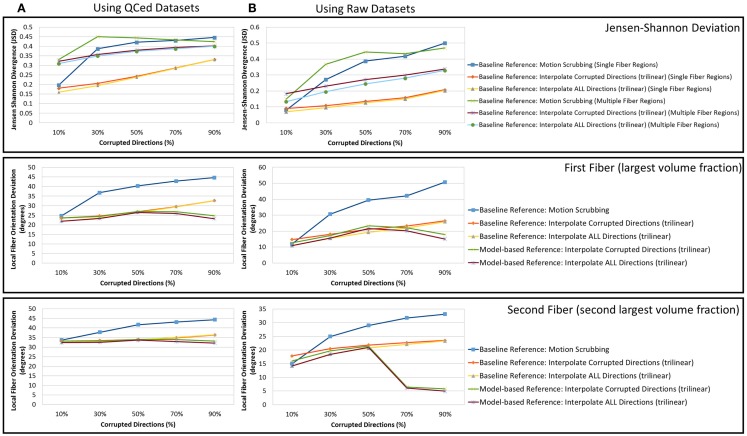
**The average Jensen–Shannon divergence (JSD) values (first row) and the average fiber orientation deviation (second and third row), a function of motion corrupted percentage for reconstructions based on gold standards generated from (A) the QCed phantom dataset and (B) the raw phantom dataset**. Note the agreement between **(A)** and **(B)** where the impact of motion scrubbing becomes more significant with more motion-corrupted directions when compared to registration-based correction. This effect is also rendered evident for local fiber orientations.

One may wonder that using a gold standard, which was obtained by motion correction (among other QC steps) using some of the methods under investigation could raise questions on reliability of the conclusions presented. Hence, in order to support the validity of the conclusions drawn from this study, we conducted the same set of experiments using the raw acquired data without performing any quality control. Figure 13 shows a sample result of the average JSD and local fiber orientation deviation metric for reconstructions based on gold standards generated from the QCed phantom datasets as well as the raw phantom datasets. Being consistent with the conclusions drawn from the reconstructions based on the QCed datasets, regions with crossing fibers are more affected by motion correction, showing larger average JSD in general when compared to the single fiber regions. The impact of motion scrubbing becomes more evident with more motion-corrupted directions when compared to the registration-based correction. Moreover, the peaked performance of the orientation deviation at 50% corrupted directions for model-based motion correction is also maintained. Further, forcing the interpolation of all gradients directions would have minimal impact on the reconstructions when compared to the choice of interpolating motion corrupted directions via setting a predefined threshold beyond which a direction is claimed to be corrupted.

### 5.1 Limitations and future work

The primary message of this paper is that care should be taken in deciding the processing pipeline for any DW-MRI (esp. HARDI) at hand, this involves, for example, the acquisition protocol (i.e., less redundant gradients would discourage the choice of motion scrubbing) and the participating subjects (i.e., elderly in pain, infants, unsedated subjects versus healthy adults where variable motion severity levels are anticipated). Nonetheless, the presented analysis attains some limitations, which can be outlined as follows:

- One-subject analysis: as a controlled motion experiment, we could use a scan session of subjects with repeated scans where the second shows bulk motion relative to the first one. The existing phantom data contain repeated scans taken in different sessions within 24 h and hence they have to be seen as independent scans for the same subject. As a pilot study, we therefore asked one healthy volunteer to be scanned twice in a single scan session while tilting the head between the two scans. This enables us to mix gradients between the two scans from the same subject; this cannot be done with the existing repeated independent scans. We understand that reporting our results with more than a pair of datasets (tilted and untilted brain) would support our analysis, and we will collect more scans with this experimental design in our future annual phantom scan sessions. Nonetheless, we think that this experiment, even with its limitations, contributes to establish an experimental framework that would guide the scientific community in systematically evaluating the outcomes of different preprocessing steps. In the future, we will prospectively plan to obtain more of such datasets, also including navigator shots for estimation of rotation, to extend this analysis.
- Anatomical geometric correction: echo-planner imaging (EPI) distortion, in contrast to Eddy current that affects only diffusion-weighted images, would affect all images in the acquired sequence regardless of their level of diffusion sensitization. Hence, EPI distortion correction would involve acquiring additional data for either B0 mapping or a dedicated T1 or T2-weighted structural target. That’s a primary reason behind ignoring EPI correction in most MRI processing pipelines (73). With the availability of such additional data, EPI correction would involve non-linear spatial warping that employ interpolation, a decision variable under investigation of the presented work. Hence, we favored to bypass this step in order not to inter-mingle interpolation due to motion correction and that of EPI correction. However, we think that the analysis/correction of inter-gradient spatial distortions, and its effect on ODF reconstruction, is an important issue, which we together with the scientific community need to address.
- Better gold standard generation: the living phantoms were healthy volunteers who were aware of the whole process and were keen to remain without motion. Nonetheless, the investigation of prospective navigators is a promising idea for future work to provide different types of ground truth data and to get motion estimates directly from the scanner rather than only via post-processing.

## Conflict of Interest Statement

The editor and reviewer Maxime Descoteaux and the reviewer Emmanuel Caruyer declare that, despite having collaborated with author Dr. Yaniv Gur, the review process was handled objectively and no conflict of interest exists. The authors declare that the research was conducted in the absence of any commercial or financial relationships that could be construed as a potential conflict of interest.

## Supplementary Material

The Supplementary Material for this article can be found online at http://www.frontiersin.org/Journal/10.3389/fneur.2014.00240/abstract

## Footnotes

^1^The protocol used a GRAPPA parallel imaging factor of 2 and a partial Fourier factor of 3/4, which does indeed result in non-Rician noise distributions. However, the effect of the noise distribution is expected to be relatively small at a b values of 2000 s/mm2 [e.g., Ref. (74)] and we do not expect the difference in noise profile to affect our conclusions in terms of the motion correction schemes.

^2^In our analysis, we opt to using a prospective approach [a Twice-Refocused Spin Echo (TRSE) sequence] for eddy current compensation in order not to introduce any alignment-based preprocessing before running motion correction that could already have a confounding effect (otherwise we would work on resampled and interpolated images before motion detection). Further, we have eye-balled the FA map of the acquired sequences (prior to motion correction) where the prominent edge artifact (regions of anomalous diffusion contrast resulted from misregistration of dissimilar materials) that should be visible in case of Eddy current distortion was almost entirely absent in the TRSE images. To further support our decision, we used FSL-MCFLIRT (31) to provide the affine transformation matrix (i.e., 12 degrees of freedom) to detect the scaling and skewing parameters, which might occur due to induced eddy current where the affine transformation matrix can be written as:

$$M=R\left(\begin{array}{ccc} s_{x} & 0 & 0\\ 0 & s_{y} & 0\\ 0 & 0 & s_{z} \end{array}\right)\left(\begin{array}{ccc} 1 & a & b\\ 0 & 1 & c\\ 0 & 0 & 1 \end{array}\right)+\left(\begin{array}{c} t_{x}\\ t_{y}\\ t_{z} \end{array}\right)$$

where *R* is the rotation matrix, *s*_x_, *s*_y_, *s*_z_ ∈ ℝ are the scaling parameters, *a, b, c* ∈ ℝ are the skewing parameters and *t*_x_, *t*_y_, *t*_z_ ∈ ℝ are the translation parameters. Table S2 in Supplementary Material reports the average and standard deviation (along all gradient directions per dataset) of the estimated transformation parameters where all datasets tend to have a unit scale with minimal skewing values. These values confirm the decision of bypassing eddy current compensation in our analysis.

^3^http://www.sci.utah.edu/software/atlaswerks.html

^4^P denotes positive and N denotes negative
