# Supplementary material for: Subject–Motion Correction in HARDI Acquisitions: Choices and Consequences
Source: Front Neurol. 2014 Dec 9;5:240. doi: 10.3389/fneur.2014.00240 (PMC4260507; doi:10.3389/fneur.2014.00240)
Supplement: Supplementary file 1 [file Table1.PDF]

Table S1 | Parcelated structures.

ROI #	Label	Region	ROI#	Label	Region
1	SPL_L	Superior parietal lobule left	89	SPL_R	Superior parietal lobule right
2	CG_L	Cingulate gyrus left	90	CG_R	Cingulate gyrus right
3	SFG_L	Superior frontal gyrus left	91	SFG_R	Superior frontal gyrus right
4	MFG_L	Middle frontal gyrus left	92	MFG_R	Middle frontal gyrus right
5	IFG_L	Inferior frontal gyrus left	93	IFG_R	Inferior frontal gyrus right
6	PreG_L	Precentral gyrus left	94	PreG_R	Precentral gyrus right
7	PoG_L	Postcentral gyrus left	95	PoG_R	Postcentral gyrus right
8	AG_L	Angular gyrus left	96	AG_R	Angular gyrus right
9	PreCu_L	Pre-cuneus left	97	PreCu_R	Pre-cuneus right
10	Cu_L	Cuneus left	98	Cu_R	Cuneus right
11	LG_L	Lingual gyrus left	99	LG_R	Lingual gyrus right
12	FuG_L	Fusiform gyrus left	100	FuG_R	Fusiform gyrus right
13	PHG_L	Parahippocampal gyrus left	101	PHG_R	Parahippocampal gyrus right
14	SOG_L	Superior occipital gyrus left	102	SOG_R	Superior occipital gyrus right
15	IOG_L	Inferior occipital gyrus left	103	IOG_R	Inferior occipital gyrus right
16	MOG_L	Middle occipital gyrus left	104	MOG_R	Middle occipital gyrus right
17	Ent_L	Entorhinal area left	105	Ent_R	Entorhinal area right
18	STG_L	Superior temporal gyrus left	106	STG_R	Superior temporal gyrus right
19	ITG_L	Inferior temporal gyrus left	107	ITG_R	Inferior temporal gyrus right
20	MTG_L	Middle temporal gyrus left	108	MTG_R	Middle temporal gyrus right
21	LFOG_L	Lateral fronto-orbital gyrus left	109	LFOG_R	Lateral fronto-orbital gyrus right
22	MFOG_L	Middle fronto-orbital gyrus left	110	MFOG_R	Middle fronto-orbital gyrus right
23	SMG_L	Supramarginal gyrus left	111	SMG_R	Supramarginal gyrus right
24	RG_L	Gyrus rectus left	112	RG_R	Gyrus rectus right
25	Ins_L	Insular left	113	Ins_R	Insular right
26	Amyg_L	Amygdala left	114	Amyg_R	Amygdala right
27	Hippo_L	Hippocampus left	115	Hippo_R	Hippocampus right
28	Cere_L	Cerebellum left	116	Cere_R	Cerebellum right
29	CST_L	Corticospinal tract left	117	CST_R	Corticospinal tract right
30	ICP_L	Inferior cerebellar peduncle left	118	ICP_R	Inferior cerebellar peduncle right
31	ML_L	Medial lemniscus left	119	ML_R	Medial lemniscus right
32	SCP_L	Superior cerebellar peduncle left	120	SCP_R	Superior cerebellar peduncle right
33	CP_L	Cerebral peduncle left	121	CP_R	Cerebral peduncle right
34	ALIC_L	Anterior limb of internal capsule left	122	ALIC_R	Anterior limb of internal capsule right
35	PLIC_L	Posterior limb of internal capsule left	123	PLIC_R	Posterior limb of internal capsule right
36	PTR_L	Posterior thalamic radiation left	124	PTR_R	Posterior thalamic radiation right
37	ACR_L	Anterior corona radiata left	125	ACR_R	Anterior corona radiata right
38	SCR_L	Superior corona radiata left	126	SCR_R	Superior corona radiata right
39	PCR_L	Posterior corona radiata left	127	PCR_R	Posterior corona radiata right
40	CGC_L	Cingulum (cingulate gyrus) left	128	CGC_R	Cingulum (cingulate gyrus) right
41	CGH_L	Cingulum (hippocampus) left	129	CGH_R	Cingulum (hippocampus) right
42	Fx/ST_L	Fornix(cres) stria terminalis left	130	Fx/ST_R	Fornix(cres) stria terminalis right
43	SLF_L	Superior longitudinal fasciculus left	131	SLF_R	Superior longitudinal fasciculus right
44	SFOF_L	Superior fronto-occipital fasciculus left	132	SFOF_R	Superior fronto-occipital fasciculus right
45	IFOF_L	Inferior fronto-occipital fasciculus left	133	IFOF_R	Inferior fronto-occipital fasciculus right
46	SS_L	Sagittal stratum left	134	SS_R	Sagittal stratum right
47	EC_L	External capsule left	135	EC_R	External capsule right
48	UNC_L	Uncinate fasciculus left	136	UNC_R	Uncinate fasciculus right
49	PCT_L	Pontine crossing tract left	137	PCT_R	Pontine crossing tract right
50	MCP_L	Middle cerebellar peduncle left	138	MCP_R	Middle cerebellar peduncle right

(Continued)

Table S1 | Continued

ROI #	Label	Region	ROI#	Label	Region
51	Fx_L	Fornix (column and body) left	139	Fx_R	Fornix right
52	GCC_L	Genu of corpus callosum left	140	GCC_R	Genu of corpus callosum right
53	BCC_L	Body of corpus callosum left	141	BCC_R	Body of corpus callosum right
54	SCC_L	Splenium of corpus callosum left	142	SCC_R	Splenium of corpus callosum right
55	RLIC_L	Retrolenticular part of internal capsule left	143	RLIC_R	Retrolenticular part of internal capsule right
56	RN_L	Red nucleus left	144	RN_R	Red nucleus right
57	SN_L	Substancia nigra left	145	SN_R	Substancia nigra right
58	Tp_L	Tapatum left	146	Tp_R	Tapatum right
59	CN_L	Caudate nucleus left	147	CN_R	Caudate nucleus right
60	P_L	Putamen left	148	P_R	Putamen right
61	Th_L	Thalamus left	149	Th_R	Thalamus right
62	GP_L	Globus pallidus left	150	GP_R	Globus pallidus right
63	MB_L	Midbrain left	151	MB_R	Midbrain right
64	Pons_L	Pons left	152	Pons_R	Pons right
65	Med_L	Medulla left	153	Med_R	Medulla right
66	SP_WM_L	Superior parietal wm left	154	SP_WM_R	Superior parietal wm right
67	CG_WM_L	Cingulum wm left	155	CG_WM_R	Cingulum wm right
68	SF_WM_L	Superior frontal wm left	156	SF_WM_R	Superior frontal wm right
69	MF_WM_L	Middle frontal wm left	157	MF_WM_R	Middle frontal wm right
70	IF_WM_L	Inferior frontal wm left	158	IF_WM_R	Inferior frontal wm right
71	Pr_WM_L	Precentral wm left	159	Pr_WM_R	Precentral wm right
72	Po_WM_L	Postcentral wm left	160	Po_WM_R	Postcentral wm right
73	A_WM_L	Angular wm left	161	A_WM_R	Angular wm right
74	PreCu_WM_L	Pre-cuneus wm left	162	PreCu_WM_R	Pre-cuneus wm right
75	Cu_WM_L	Cuneus wm left	163	Cu_WM_R	Cuneus wm right
76	L_WM_L	Lingual wm left	164	L_WM_R	Lingual wm right
77	Fu_WM_L	Fusiform wm left	165	Fu_WM_R	Fusiform wm right
78	SO_WM_L	Superior occipital wm left	166	SO_WM_R	Superior occipital wm right
79	IO_WM_L	Inferior occipital wm left	167	IO_WM_R	Inferior occipital wm right
80	MO_WM_L	Middle occipital wm left	168	MO_WM_R	Middle occipital wm right
81	ST_WM_L	Superior temporal wm left	169	ST_WM_R	Superior temporal wm right
82	IT_WM_L	Inferior temporal wm left	170	IT_WM_R	Inferior temporal wm right
83	MT_WM_L	Middle temporal wm left	171	MT_WM_R	Middle temporal wm right
84	LFO_WM_L	Lateral fronto-orbital wm left	172	LFO_WM_R	Lateral fronto-orbital wm right
85	MFO_WM_L	Middle fronto-orbital wm left	173	MFO_WM_R	Middle fronto-orbital wm right
86	SM_WM_L	Supramarginal wm left	174	SM_WM_R	Supramarginal wm right
87	Rect_WM_L	Rectus wm left	175	Rect_WM_R	Rectus wm right
88	Cere_WM_L	Cerebellum wm left	176	Cere_WM_R	Cerebellum wm right
