# Supplementary material for: Subject–Motion Correction in HARDI Acquisitions: Choices and Consequences
Source: Front Neurol. 2014 Dec 9;5:240. doi: 10.3389/fneur.2014.00240 (PMC4260507; doi:10.3389/fneur.2014.00240)
Supplement: Supplementary file 2 [file Table2.PDF]

Table S2 | The average and standard deviation of the affine transformation parameters.

Dataset ID	Translation vector magnitude (mm)	Rotation angle (degrees)	Scale in *x* direction	Scale in *y* direction	Scale in *z* direction	Skew A	Skew B	Skew C
ine phan1_time1_chop	0.263283 ± 0.129780	0.4258 ± 0.1765	0.974387 ± 0.122766	0.991650 ± 0.125002	0.978987 ± 0.123368	−0.001053 ± 0.001162	−0.000104 ± 0.005986	−0.000753 ± 0.001325
Phan1_time1_unc_hos	0.470658 ± 0.232117	0.1534 ± 0.0918	0.967640 ± 0.120958	0.987012 ± 0.123419	0.969454 ± 0.121189	−0.002346 ± 0.001614	0.001971 ± 0.000873	−0.002854 ± 0.002759
Phan1_time1_unc_res	0.189921 ± 0.063812	0.2667 ± 0.0947	0.969042 ± 0.121136	0.981819 ± 0.122801	0.968367 ± 0.121047	0.001694 ± 0.001812	−0.000951 ± 0.001204	−0.003887 ± 0.002544
Phan1_time1_washu_res	0.592816 ± 0.167149	0.0998 ± 0.108	0.972356 ± 0.122509	0.985866 ± 0.124311	0.980857 ± 0.123619	−0.000613 ± 0.001537	0.001895 ± 0.001171	0.000042 ± 0.002984
Phan1_time2_chop	0.476715 ± 0.158225	0.2922 ± 0.1342	0.970214 ± 0.122241	0.981341 ± 0.123652	0.974277 ± 0.122824	−0.004799 ± 0.002259	0.001672 ± 0.001119	−0.004175 ± 0.002521
Phan1_time2_unc_hos	0.580458 ± 0.205031	0.1544 ± 0.0845	0.968968 ± 0.121126	0.985392 ± 0.123211	0.969308 ± 0.121165	−0.000668 ± 0.001327	0.003067 ± 0.001256	−0.003470 ± 0.001561
Phan1_time2_unc_res	0.282364 ± 0.103415	0.3059 ± 0.186	0.966004 ± 0.120753	0.974620 ± 0.121831	0.967014 ± 0.120879	−0.000146 ± 0.000979	0.001095 ± 0.000984	0.001062 ± 0.001672
Phan1_time2_washu_res	0.519812 ± 0.201197	0.1394 ± 0.1097	0.971916 ± 0.124449	0.988555 ± 0.126727	0.974220 ± 0.124811	−0.001642 ± 0.001679	−0.001064 ± 0.001624	−0.000475 ± 0.002170
Phan2_time1_chop	0.476104 ± 0.150039	0.2744 ± 0.2404	0.973545 ± 0.124653	0.987570 ± 0.126611	0.977957 ± 0.125338	−0.000196 ± 0.001119	0.002787 ± 0.000843	0.000057 ± 0.002648
Phan2_time1_unc_hos	0.310917 ± 0.145060	0.1143 ± 0.1271	0.973924 ± 0.124700	0.989896 ± 0.126798	0.972212 ± 0.124521	0.001524 ± 0.001987	0.001644 ± 0.003226	−0.001149 ± 0.002316
Phan2_time1_unc_res	0.573942 ± 0.159912	0.338 ± 0.2473	0.972004 ± 0.125488	0.992603 ± 0.128153	0.978598 ± 0.126395	−0.001017 ± 0.000745	−0.001052 ± 0.001142	0.000181 ± 0.001112
Phan2_time1_washu_res	0.399943 ± 0.159193	0.2744 ± 0.1721	0.973515 ± 0.122656	0.990590 ± 0.124862	0.978706 ± 0.123327	0.001399 ± 0.001533	−0.000429 ± 0.002762	−0.002402 ± 0.002564
Phan2_time2_chop	0.334249 ± 0.148992	0.5177 ± 0.2077	0.975330 ± 0.124881	0.986590 ± 0.126375	0.977786 ± 0.125299	0.000482 ± 0.001227	0.000831 ± 0.001404	0.001103 ± 0.002349
Phan2_time2_unc_hos	0.657453 ± 0.159169	0.551 ± 0.1521	0.972614 ± 0.122543	0.986810 ± 0.124331	0.979568 ± 0.123450	0.000448 ± 0.001303	0.001030 ± 0.000899	0.001600 ± 0.002339
Phan2_time2_unc_res	0.166599 ± 0.063907	0.4218 ± 0.231	0.971802 ± 0.122443	0.982748 ± 0.123822	0.969650 ± 0.122169	0.007760 ± 0.001445	0.001021 ± 0.001453	−0.000299 ± 0.001389
Phan2_time2_washu_res	0.201228 ± 0.058342	0.4382 ± 0.1562	0.973766 ± 0.123671	0.983821 ± 0.124961	0.972836 ± 0.123570	−0.002218 ± 0.001863	−0.001634 ± 0.002000	−0.001353 ± 0.002329
Phan3_time1_chop	0.259133 ± 0.175041	0.6439 ± 0.126	0.971986 ± 0.123448	0.986653 ± 0.125336	0.976689 ± 0.124069	0.001603 ± 0.001702	−0.004551 ± 0.002228	0.003174 ± 0.004675
Phan3_time1_sea	0.452670 ± 0.297523	0.3769 ± 0.2054	0.988928 ± 0.127704	0.991992 ± 0.128127	0.989708 ± 0.127798	0.003240 ± 0.002314	0.000590 ± 0.001049	0.000753 ± 0.001993
Phan3_time1_unc_hos	0.635246 ± 0.158109	0.1311 ± 0.127	0.975263 ± 0.122875	0.989361 ± 0.124665	0.982201 ± 0.123788	0.001640 ± 0.001893	0.001942 ± 0.000933	0.000695 ± 0.003627
Phan3_time1_unc_res	0.539050 ± 0.152979	0.4504 ± 0.1164	0.973626 ± 0.122668	0.987238 ± 0.124383	0.979584 ± 0.123445	0.001921 ± 0.001540	0.004630 ± 0.002217	0.000202 ± 0.002536
Phan3_time2_chop	0.280374 ± 0.086683	0.1584 ± 0.1157	0.973434 ± 0.123631	0.984695 ± 0.125073	0.972197 ± 0.123512	0.002663 ± 0.002568	0.003467 ± 0.001624	0.000037 ± 0.002711
Phan3_time2_sea	0.556044 ± 0.238928	0.2208 ± 0.133	0.988590 ± 0.127661	0.992080 ± 0.128118	0.984150 ± 0.127070	0.002273 ± 0.001335	0.000950 ± 0.000859	0.001297 ± 0.002274
Phan3_time2_unc_hos	0.526151 ± 0.216508	0.1569 ± 0.1836	0.974415 ± 0.122768	0.990469 ± 0.124820	0.980314 ± 0.123656	−0.001155 ± 0.002091	0.002274 ± 0.000998	0.003932 ± 0.002886
Phan3_time2_unc_res	0.743160 ± 0.172370	0.1276 ± 0.0877	0.972536 ± 0.124526	0.986215 ± 0.126279	0.981740 ± 0.125742	−0.000120 ± 0.001058	0.002701 ± 0.001266	0.002270 ± 0.004077
